# Development of silver-based hybrid nanoparticles loaded with eEF2 K-siRNA and quercetin against triple-negative breast cancer

**DOI:** 10.1007/s13346-025-01860-6

**Published:** 2025-04-23

**Authors:** Orhan Burak Eksi, Ahsen Guler, Munevver Akdeniz, Pinar Atalay, Zuhal Hamurcu, Omer Aydin

**Affiliations:** 1 ERNAM-Nanotechnology Research and Application Center, Erciyes University, Kayseri, 38039 Turkey; 2 NanoThera Lab, ERFARMA-Drug Application and Research Center, Erciyes University, Kayseri, 38039 Turkey; 3 Department of Medical Biology, Faculty of Medicine, Erciyes University, Kayseri, 38039 Turkey; 4 GENKOK-Betül-Ziya Eren Genome and Stem Cell Center, Erciyes University, 38039 Kayseri, Turkey; 5 Biomedical Engineering, Erciyes University, Kayseri, 38039 Turkey; 6 Department of Nanomedicine, Houston Methodist Research Institute, Houston, TX 77030 USA; 7 ERKAM-Clinical Engineering Research and Implementation Center, Erciyes University, 38030 Kayseri, Turkey

**Keywords:** Triple Negative Breast Cancer (TNBC), SiRNA, EEF2 K, Quercetin, Hybrid Nanoparticles (HNP), Laber-by-layer

## Abstract

Breast cancer is the most common cancer among women, with approximately 2.3 million new cases globally. Triple-negative breast cancer (TNBC) is an aggressive subtype characterized by the lack of estrogen receptor (ER), progesterone receptor (PR), and HER2 expression, making it unresponsive to traditional therapies. Eukaryotic Elongation Factor 2 Kinase (eEF2K) is overexpressed in TNBC, promoting cell survival by inhibiting apoptosis through phosphorylation of eEF2. Recently, eEF2K has been targeted for cancer therapy, and siRNA-based gene therapy has emerged as an effective approach to silence overexpressed genes. However, siRNA delivery is challenging due to its instability and susceptibility to degradation. In this study, we developed a novel hybrid nanoparticle (HNP) using a Layer-by-Layer (LbL) method for siRNA delivery targeting eEF2K in TNBC. The HNPs consist of a silver nanoparticle (AgNP) core, coated with poly (allylamine hydrochloride) (PAH) and poly(styrene sulfonate) (PSS), and loaded with eEF2K-siRNA and quercetin (QU), a chemotherapeutic agent, in separate layers. The nanoparticles also incorporated 4-ATP molecules for Raman traceability. In vitro experiments on TNBC cell lines (MDA-MB-231, BT-549, 4T1) showed that the combination therapy of eEF2K-siRNA and QU reduced cell viability, inhibited colony formation, and suppressed cell migration. At high 120 nM of siRNA concentration, 3D spheroid disintegration, activation of apoptotic pathways, and eventual necrotic cell death were observed. The results demonstrate that the developed HNPs are non-toxic, effective, and offer potential as a theranostic platform for TNBC treatment.

**Graphical Abstract:**

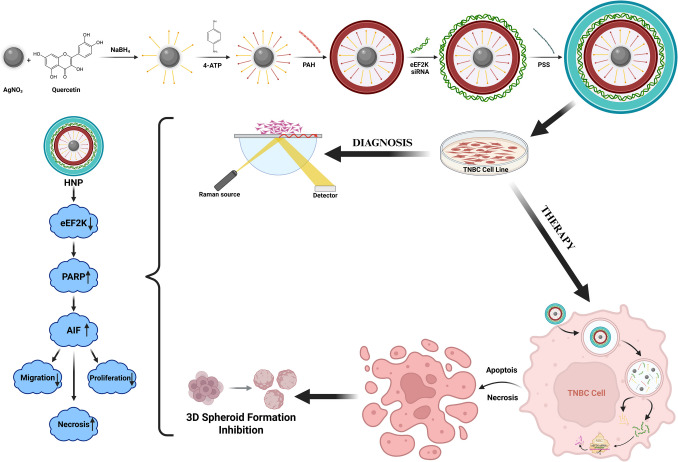

**Supplementary Information:**

The online version contains supplementary material available at 10.1007/s13346-025-01860-6.

## Introduction

Breast cancer is the most common cancer in women worldwide. According to reported data, there are approximately 2.3 million new cases of breast cancer on the global scale. These numbers are on the rise, making breast cancer a widespread health concern globally [1]. There are many subtypes of breast cancer, and triple-negative breast cancer (TNBC) is one of them. TNBC is characterized by the absence or suppression of estrogen receptor (ER), progesterone receptor (PR), and human epidermal growth factor receptor 2 (HER2) expression. TNBC accounts for approximately 15–20% of all breast cancer cases and is more aggressive than other types of breast cancer due to the absence of these receptors, making it less responsive to hormone therapy, chemotherapy, or HER2-targeted therapies [2].

Eukaryotic Elongation Factor 2 Kinase (eEF2 K) is a Ca^2^⁺/calmodulin-dependent Ser/Thr kinase that functions as a negative regulator of protein synthesis by phosphorylating Eukaryotic Elongation Factor 2 (eEF2). The only known substrate of eEF2 K is eEF2, which plays a crucial role in ribosomal translocation from the A-site to the P-site during protein translation. Phosphorylation of eEF2 by eEF2 K impairs its binding to the ribosome, thereby reducing the elongation rate and inhibiting protein synthesis. In TNBCs, eEF2 K is overexpressed, contributing to tumor progression and poor prognosis. While eEF2 functions as a cell division marker, eEF2 K regulates this system by converting eEF2 into its inactive, phosphorylated form. Although phosphorylated eEF2 remains in the cell, it cannot initiate division, thereby maintaining cellular stability [3]. Recent studies have also shown that eEF2 K is overexpressed in various other solid tumors, including pancreatic cancer, colon cancer, glioblastoma, and breast cancer, making it a potential therapeutic target in cancer treatment. [4].

It is well-known that tumor tissues and the surrounding cancerous regions are nutrient-poor environments [5, 6]. Due to the aggressive nature of cancer cells, their metabolic activities are rapid, requiring significantly more energy than normal cells. This leads to intercellular competition for limited nutrients, causing cellular starvation and energy deficiency, which can ultimately result in cell death. To survive under these harsh conditions, breast cancer cells adapt by activating eEF2 K, which phosphorylates and inactivates eEF2, halting cell division. This conserves energy by preventing high-energy activities like protein synthesis and DNA replication, enabling cancer cells to endure in nutrient-deprived environments [7]. Additionally, it has been shown that eEF2 K plays a role in regulating apoptosis. Due to its effect, eEF2 K is overexpressed in many tumor types, including breast cancer, and this overexpression is associated with poor prognosis and resistance to treatment [4, 8, 9].

Studies have shown that eEF2 K promotes cell survival by suppressing apoptosis. This occurs through the phosphorylation of eEF2, resulting in decreased activity in a protein called BAD. BAD is a pro-apoptotic protein that inhibits the activity of anti-apoptotic proteins like Bcl- 2, promoting cell death [8].

Recently, eEF2 K has been actively investigated as a therapeutic target for breast cancer, and many eEF2 K inhibitors, such as NH125, TX- 1918, A- 484954, and fluoxetine, etc., have been developed. These inhibitors are designed to block eEF2 K activity and reduce eEF2 phosphorylation. This would activate pro-apoptotic proteins and initiate apoptosis [4, 9]. Among all inhibitors, siRNA-based gene therapy is the most effective due to the alternative method to overcome TNBC rather than hormone therapy, chemotherapy, etc.

In this approach, gene expression and mutations occurring at the post-transcriptional level are managed, reprogrammed, or silenced. After the cells take up siRNA molecules, they are incorporated into the RNA-induced silencing complex (RISC), forming a stable interaction. This allows the siRNA molecules to be delivered to the targeted mRNA. Specifically, sequenced siRNA molecules bind to the mRNA, and the targeted mRNA is degraded with the help of RISC proteins. In this manner, overexpressed genes or proteins would be silenced at the post-transcriptional level [10].

Even though siRNA-based gene therapy is a promising treatment for cancer, its application faces significant challenges, particularly in the efficient delivery of siRNA molecules. Naked siRNA, due to its small size, is susceptible to several barriers in the body. Once systemically injected, siRNA drugs first encounter extracellular obstacles, including enzymatic degradation by endonucleases and RNases, as well as repulsion by the negatively charged cell membrane. Additionally, the immune system recognizes siRNA molecules as exogenous, which can trigger immune responses. Specifically, Toll-like receptors (TLRs) and Retinoic Acid Inducible Gene-I-like Receptors (RLRs) on immune cells can detect the presence of siRNA, leading to the activation of immune pathways and inflammation. These factors, combined with the inherent instability of siRNA, make overcoming these challenges particularly difficult. As a result, a nanodelivery system is essential to protect siRNA from degradation, enhance cellular uptake, and reduce immunogenic responses, thus ensuring the effective delivery of siRNA for gene therapy applications [10–13].

Recently, many delivery systems have been tested to carry out gene therapy successfully. In this case, copolymer-based micelles, charged lipid-based nanoparticles, liposomal formulations, and metallic nanoparticles have been used as traditional delivery systems [10]. However, several factors must be overcome to develop effective siRNA therapeutics for systemically treating advanced diseases. Firstly, it is ensured that nanoparticles carrying siRNA remain in the bloodstream long enough to reach tumor sites, leading to sustained treatment. Also, high enough siRNA must be loaded into the delivery system to compensate for the low cellular uptake and low possibility of endosomal escape. Lastly, the materials used in the delivery system must facilitate to escape of siRNA molecules from endosomes while minimizing the toxicity [14, 15]. Although the traditional delivery systems could fulfill some of those requirements, unfortunately, they have some obstacles to handle. For example, it is hard to control the siRNA loading side in traditional delivery systems. Additionally, there is a toxic side effect while arranging the optimum dosage for siRNA therapeutic. Finally, those delivery systems are unsuitable for applying a combination therapy on a single delivery platform. Combining gene therapy and chemotherapy is crucial for some cases so arranging an optimum combination level must be necessary [16].

Layer-by-Layer (LbL) nanoparticles utilize oppositely charged components assembled in successive layers, offering a highly tunable and modular nanodelivery platform. Compared to conventional nanoparticle synthesis methods such as single-step self-assembly, emulsification, or co-precipitation, the LbL approach provides greater control over particle architecture, allowing for the sequential deposition of different materials with well-defined thicknesses and compositions. This precise layering enables the incorporation of multiple therapeutic agents, including chemotherapeutics, gene silencing molecules (e.g., siRNA), and targeting ligands, in distinct compartments, thereby facilitating co-delivery or sequential release strategies for synergistic therapeutic effects. Furthermore, LbL nanoparticles allow for diverse interaction mechanisms, including electrostatic attraction [17], hydrogen bonding [18], and covalent cross-linking [19], providing enhanced structural stability and tunability compared to conventional encapsulation methods. The well-ordered multilayered structure offers advantages in surface functionalization, enabling the attachment of imaging probes, targeting moieties, or stimuli-responsive coatings to improve bioavailability and reduce off-target toxicity. Given these attributes, the LbL technique is a versatile and promising alternative to traditional nanoparticle fabrication methods in advanced drug and gene delivery applications [16, 20–22].

**Fig. 1:**
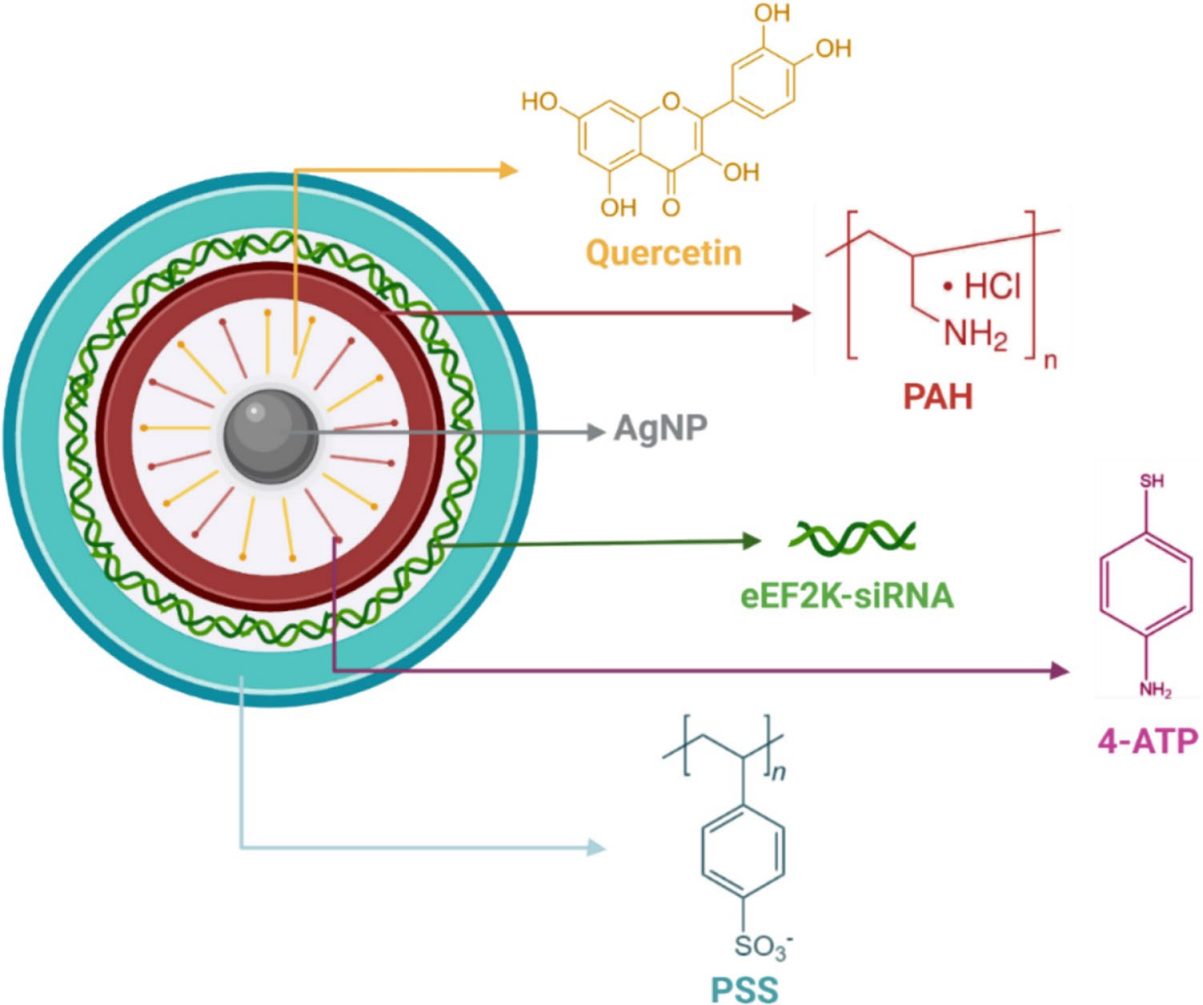
The illustration of the engineered HNP components containing of quercetin (inducer), PAH (positively charged polymer), eEF2 K siRNA (gene therapy agent), PSS (negatively charged polymer), and 4-ATP (Raman-active agent)

In this study, we sought the effect of eEF2 K-siRNA by supporting quercetin (QU) molecules, known as a chemotherapeutic agent in a novel hybrid nanoparticle (HNP) developed as applying LbL method, on TNBC-type breast cancer (Fig. 1). The production of HNP possesses electrostatic interactions between each layer by covering a silver nanoparticle (AgNP) core with a positively charged polymer called poly(allylamine hydrochloride) (PAH) and a negatively charged polymer called poly(styrene sulfonate) (PSS), sequentially. In this manner, eEF2 K-siRNA and QU molecules were loaded into different layers from each other, so we observed the effect of each therapeutic on TNBC cell lines (MDA-MB- 231, BT- 549, and 4T1), individually and simultaneously. Furthermore, due to the allowance of the LbL method, HNPs were conjugated with 4-ATP molecules at the AgNP core. Since 4-ATP molecules are Raman active molecules, our developed nanoparticles were able to be traceable under Raman spectroscopy, and in this way, they gained a theranostic property. After completing the production of HNP with gene therapy and chemotherapy agent, we performed in vitro experiments to examine the effect on TNBC. The results demonstrated that the developed particle was non-toxic, and that the combination therapy reduced cell viability, inhibited colony formation, and suppressed cellular migration. Moreover, at a high siRNA concentration, it was observed that 3D spheroids were disintegrated, apoptotic cell pathways were activated, but the cells were ultimately killed through necrosis.

## Material & methods

### Materials

Silver nitrate (AgNO₃, Merck, cat# 7761–88 - 8), quercetin (Sigma-Aldrich, cat# Q4951 - 100G), sodium hydroxide solution (NaOH, 1 M, 10 mL, Sigma-Aldrich, cat# S5881 - 500G), and sodium borohydride (NaBH₄, 40 mM, 10 mL, Fluka, cat# 71,321 - 100G) were used in the synthesis processes. Deionized water (dH₂O) was produced in the laboratory. For surface functionalization and layer-by-layer assembly, 4-aminothiophenol (4-ATP, Sigma-Aldrich, cat# 422,967 - 5G), poly(allylamine hydrochloride) (PAH, Thermo Scientific, cat# 11,384,748), and poly(styrene sulfonate) (PSS, Thermo Scientific, cat# 10,328,550) were utilized. The anti-eEF2 K siRNA (SASI_Hs01_00060065, 100 µM) was synthesized with the following nucleotide sequences: sense strand 5’-CUCAUCACAUCCUAGCCGA[dT][dT]− 3’ and antisense strand 5’-UCGGCUAGGAUGUGAUGAG[dT][dT]− 3’. Triple-negative breast cancer (TNBC) cell lines including MDA-MB- 231, BT- 549, and 4T1 were cultured in Dulbecco’s Modified Eagle Medium (DMEM, Merck, cat# D6429 - 500ML) and passaged using Trypsin–EDTA (Merck, cat# T4049 - 100ML). For cellular tracking and transfection efficiency, fluorescently labeled (FAM) anti-GAPDH siRNA (Thermo Scientific, cat# AM4650) and DAPI staining solution (Sigma-Aldrich, cat# 10236276001) were used. Cell viability and apoptosis analyses were performed using resazurin (Thermo Fisher Scientific, cat# R12204) and the Annexin V/PI staining kit (Thermo Scientific, cat# V13242), respectively.

### eEF2 K expression in human breast cancer and Kaplan–Meier survival analysis

The prognostic significance of eukaryotic elongation factor 2 kinase (eEF2 K) expression in breast cancer was assessed using the Kaplan–Meier Plotter online platform (http://kmplot.com), which integrates gene expression and survival data from multiple public repositories, including GEO, EGA, and TCGA.

For this study, the breast cancer (BRCA) cohort was selected from the mRNA gene chip dataset, and either overall survival (OS) or relapse-free survival (RFS) was used as the clinical endpoint, depending on the analysis. Patients were automatically stratified into high and low eEF2 K expression groups based on tertile distribution, with the top third (66 th–100 th percentile) classified as high expression and the bottom third (0–33rd percentile) as low expression.

Kaplan–Meier survival curves were generated to visualize survival differences between groups, and statistical significance was determined using the log-rank test. Hazard ratios (HRs) and 95% confidence intervals (CIs) were calculated to quantify the risk associated with high eEF2 K expression. The number of patients included in each group and those at risk at specific time points were displayed beneath the survival plots.

Additional subgroup analyses were performed within clinically relevant breast cancer subtypes, including triple-negative breast cancer (TNBC) (ER −, PR −, HER2 −), PAM50-defined basal-like subtype, and node-positive basal tumors, where available. All analyses were conducted using the default parameters and interface tools provided by the KM Plotter platform.

### Hybrid nanoparticle synthesis

#### Synthesis of silver nanoparticles assisted by quercetin

Initially, 18 mg of AgNO_3_ (Merck, cat# 7761–88 - 8) was weighed and added into a vial to prepare an aqueous solution. Subsequently, 20 mg of quercetin powder (Sigma-Aldrich, cat# Q4951 - 100G) was dissolved in a NaOH solution (1 M, 10 mL) (Sigma-Aldrich, cat# S5881 - 500G) and added dropwise to the AgNO_3_ solution under stirring. The mixture was then incubated at room temperature on a magnetic stirrer for 5 min to further reduce the size of the silver nanoparticles (AgNPs). Following this, an aqueous solution of NaBH_4_ (40 mM, 10 mL) (Fluka, cat# 71,321 - 100G) was added to the mixture to promote further reduction, and the final volume was adjusted to 5 mL with deionized water (dH_2_O). The mixture was then incubated for 2 h, leading to the formation of AgNPs.

**Fig. 2:**
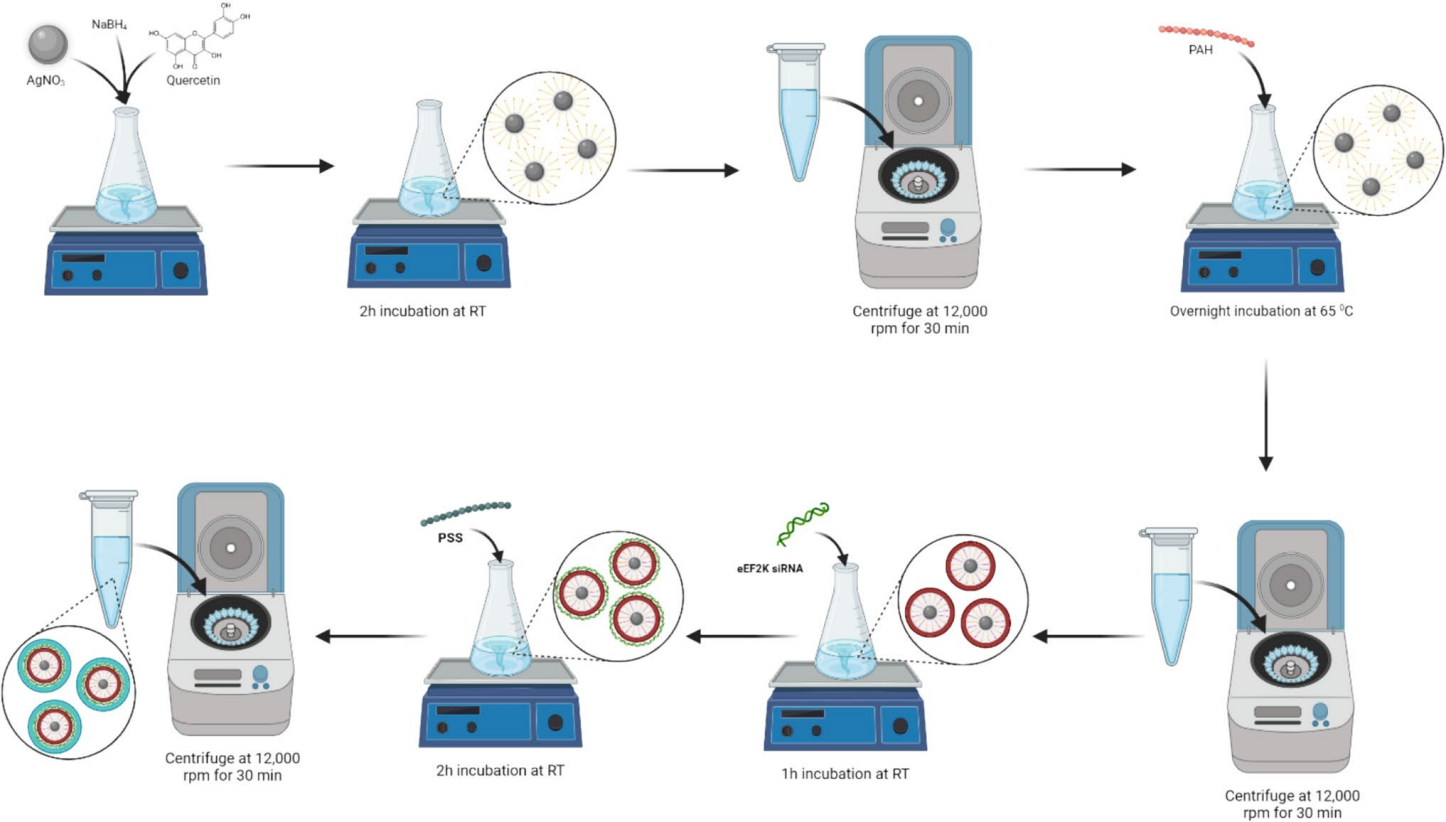
The procedure of the hybrid nanoparticles produced by LbL method

Subsequently, the AgNP + QU particles were purified to remove free quercetin molecules and unreacted silver salts. Initially, the nanoparticles were pelleted by centrifugation at 15,000 rpm for 20 min, and the supernatant was discarded. The pellet was then resuspended in dH2O. The washing step was repeated by centrifuging the resuspended nanoparticles again at 15,000 rpm for 20 min. Finally, the resulting pellet was resuspended in 1 mL of dH_2_O to obtain a usable form of the nanoparticles [23]. Each step of the synthesis is shown in Fig. 2.

#### Determining the loading efficiency of quercetin onto AgNP

After the synthesis of the developed HNP particles was completed, standards of quercetin molecules were prepared at different concentrations (0.125, 0.25, 1, and 2 mM) to determine the amount of quercetin bound to the AgNP core. By scanning the UV–Vis region (200–900 nm), the maximum absorbance wavelength of quercetin molecules was identified as 371 nm. The absorbance of each standard solution was then measured at 371 nm. Based on the results, an absorbance-concentration calibration curve was plotted in GraphPad, and the linear regression equation and R^2^ value were calculated.

#### 4-ATP conjugation of the synthesized AgNP + QU complex

4.15 mg of 4-ATP (Raman-active molecule) (Sigma Aldrich, cat# 422,967 - 5G) was weighed and dissolved in 1 mL of dH2O. It is then mixed with the previously synthesized 1 mL of AgNP + QU. The total volume was adjusted to 5 mL with dH2O, and the mixture was incubated on a magnetic stirrer at 65 °C for 12 h, followed by washing as in the AgNP + QU synthesis. This allows for the forming of sulfur bonds between thiol groups (-SH) of 4-ATP and the silver nanoparticles, completing and purifying the synthesis [24].

#### Coating of the synthesized AgNP + QU + 4-ATP particles with Poly(allylamine hydrochloride) (PAH)

3.87 mg of PAH (Thermo Scientific cat# 11,384,748) was weighed and dissolved in 1 mL of dH2O. It was then mixed with 667 µL of AgNP + QU + 4-ATP, and the total volume was adjusted to 5 mL with dH2O. The mixture was incubated on a magnetic stirrer at room temperature for 3 h. Finally, washing was performed as in the AgNP + QU synthesis. At this stage, the outer layer of the system was coated with a positively charged polymer, PAH, preparing the system for genetic material incorporation [25].

#### Loading of eEF2 K-siRNA onto the synthesized AgNP + QU + 4-ATP + PAH hybrid nanoparticle

227 µL of the product prepared in the previous step was mixed with 1.25 µL of 100 µM anti-eEF2 K siRNA (SASI_Hs01_00060065), and the total volume was adjusted to 500 µL. The system was then incubated on an orbital shaker at room temperature for 1 h. This allows for electrostatic interaction between the phosphate groups of the genetic materials and the positively charged shell due to PAH.

Nucleotide sequence of the eEF2 K-siRNA (SASI_Hs01_00060065) molecule:

- **Sense strand:** 5’-CUCAUCACAUCCUAGCCGA [dT][dT]− 3’
- **Antisense strand:** 5’-UCGGCUAGGAUGUGAUGAG [dT][dT]− 3’

#### Coating of eEF2 K-siRNA loaded HNPs with Poly(styrene sulfonate) (PSS)

2.7 mg of PSS (Thermo Scientific, cat# 10,328,550) was weighed and dissolved in 1 mL of dH2O. The entire 500 µL of particles synthesized in the previous step was mixed, and the total volume was adjusted to 5 mL. Subsequently, incubation was carried out on a magnetic stirrer at room temperature for 3 h, followed by washing as in the AgNP + QU synthesis. After this stage, negatively charged PSS molecules were electrostatically attached to the positively charged surface due to PAH, rendering the particle entirely biocompatible.

### Characterization of HNP

After synthesis, the size distribution and concentration of full complex hybrid nanoparticles (HNPs) were analyzed using a nanoparticle tracking analyzer (NTA) (Malvern Panalytical, NanoSight NS300). These measurements provide insights into the uniformity and stability of the nanoparticles, which are crucial for their biological interactions and therapeutic efficacy. To determine the encapsulation efficiency of Quercetin, a standard calibration curve was obtained using different concentrations of quercetin standards, measured with a UV/Vis spectrometer (PerkinElmer) at 371 nm. Morphological analysis of both unmodified and modified HNPs was performed using scanning transmission electron microscopy (STEM; Zeiss) to assess structural integrity, which plays a role in cellular uptake and drug release. Since all synthesis steps rely on electrostatic interactions, zeta potential analysis used a Zetasizer Nano ZS (Malvern) to evaluate surface charge variations, influencing nanoparticle stability. Furthermore, elemental composition was examined using Energy-dispersive X-ray spectroscopy (EDX; Zeiss) to confirm the presence and distribution of key therapeutic and structural elements, ensuring the integrity of the formulation. By integrating these characterization techniques, we aimed to comprehensively assess the physicochemical properties of HNPs, which directly impact their therapeutic potential.

### In vitro experiments

This study used TNBC cell lines: MDA-MB- 231, BT- 549, and 4T1. MDA-MB- 231 and BT- 549 are human TNBC cell lines, while 4T1 is a mouse cell line. The cells were cultured in an incubator at 37 °C, with 95% humidity and 5% CO2, in DMEM medium supplemented with 10% fetal bovine serum (FBS), 100U/mL penicillin, and 100U/mL streptomycin. When the cells reached 80% confluency, they were passaged using trypsin–EDTA, an enzyme that detaches adherent cells from the surface. The detached cells were centrifuged at 1500 rpm for 5 min and then reseeded.

#### Cellular uptake

Cells were seeded onto glass-bottom single wells at a density of 400,000 cells per well and incubated overnight to allow attachment to the surface. The cells were then treated with hybrid nanoparticles loaded with fluorescently labeled (FAM) anti-GAPDH siRNA and incubated for 6 h. After incubation, the cells were washed with PBS and fixed with 4% paraformaldehyde for 15 min to ensure surface fixation. After fixation, the cells were rewashed with PBS, followed by DAPI staining for 15 min to highlight the nuclei under fluorescent imaging. After another PBS wash, the cells were prepared for imaging and analyzed using a Leica & Dm Il Led Fluo fluorescent inverted microscope adjusting 20X magnification, 30 ms exposure, and using DAPI (Ex: 350–380 nm, Em:420–480 nm) and FITC (Ex:450–490 nm, Em:515–555 nm) filters [26].

#### Cellular viability

Cell viability was determined using the resazurin assay according to a previously established procedure. Cells were seeded into 96-well plates at a concentration of 1 × 10^4^ cells per well and incubated overnight for attachment. After incubation, the adhered cells were treated with hybrid nanoparticles at various concentrations (1.88, 3.75, 7.5, 15, and 30 nM) for 24, 48, and 72 h. Following the designated incubation periods, the supernatant was removed, and resazurin (Thermo Fisher Scientific, cat# R12204) was added to each well. After a 3-h incubation, absorbance measurements were taken using a Promega Glomax ELISA reader under a green filter [26].

#### Colony formation assay

The cells were seeded into 12-well plates at a density of 750 cells per well, and HNPs containing siRNA at concentrations of 3.75, 7.5, 15 and 30 nM were added. After 13 days, the medium containing the HNPs was removed, and the cells were stained with 1% crystal violet in 10% methanol. Following a 10-min incubation at room temperature, the mixture was discarded, and the wells were washed three times with PBS to remove excess crystal violet. Colonies containing 50 or more cells were counted and analyzed using ImageJ software [27].

#### Wound healing (Migration) assay

To investigate the suppression of cancer cell metastatic capability, the wound healing assay was employed following the previous studies [28]. The cells were seeded onto 24-well plates at a density of 250.000 cells/well, allowing cells to cover 95% of the wells. After incubation for surface adherence, a scratch was made in the middle of the wells using a 200 µL pipette tip, termed as the"wound". Cells passing over this scratch were removed, and the wells were washed with PBS to eliminate any remaining cells and debris. After 72 h of incubation, analysis will be performed using ImageJ.

#### Apoptosis assay

Cells were seeded into 12-well plates at a density of 100,000 cells per well and incubated overnight. The next day, the cells were treated with hybrid nanoparticles at various concentrations (1.88, 3.75, 7.5, 15, and 30 µM) for 72 h. At the end of the incubation, both dead and live cells were collected. Live cells were detached from the surface using trypsin–EDTA, and after removal of the enzymes by centrifugation at 1500 rpm for 5 min at 24 °C, the supernatant was discarded, and the cell pellets were resuspended in 300 µL PBS. Subsequently, Annexin V and PI staining was performed, and apoptosis analysis was conducted using the Milipore Guava easyCyte 5 flow cytometer [29].

#### Protein level analysis

Cells were seeded into T25 flasks at a density of 350,000 cells per flask and incubated overnight. They were then treated with hybrid nanoparticles at varying concentrations (1.88, 3.75, 7.5, 15, and 30 nM) for 72 h. After incubation, cells were harvested, washed twice with ice-cold PBS, and lysed in a lysis buffer at 4 °C. Protein concentrations were measured using a protein assay kit (DC kit; Bio-Rad, Hercules, CA). A total of 40 μg of protein from each sample was separated by sodium dodecyl sulfate–polyacrylamide gel electrophoresis (SDS-PAGE) on a 4–20% gradient gel and transferred onto polyvinylidene difluoride (PVDF) membranes. The membranes were blocked with 5% dry milk in Tris-buffered saline–Tween 20 (TBS-T) for 60 min. After washing with TBS-T, the membranes were incubated with the following primary antibodies: eEF2 K (Cell Signaling, cat# 3692S), caspase- 3 (Proteintech, cat# 19,677–1-AP), cleaved caspase- 3 (Elabscience, cat# E-AB- 30004), AIF (Cell Signaling, cat# D39D2), PARP (Proteintech, cat# 13,371–1-AP), and β-actin (Proteintech, cat# 60,008–1-Ig). Following another wash with TBS-T, the membranes were incubated with horseradish peroxidase-conjugated anti-rabbit (Bio-Rad, #170–6515) or anti-mouse secondary antibodies (Bio-Rad). All antibodies were diluted in TBS-T containing 5% dry milk. Detection was carried out using the Clarity Western ECL Substrate (Bio-Rad), and blots were visualized with the ChemiDoc MP Imaging System (Bio-Rad). A housekeeping protein such as β-actin was used as a loading control with the assumption that the expression levels of these proteins remain constant. Quantitating a western blot analysis refers to measuring the signal emitted by our target protein band(s). The signal intensity of the target protein band was normalized with the signal intensity of the housekeeping protein band. Densitometric quantification was performed using the ChemiDoc MP Imager software (Bio-Rad) [28].

#### Testing of developed HNPs in 3D triple-negative breast tumor models

Solutions of 5.0% (w/w) polyethylene glycol (PEG, 35 kDa, Sigma) and 12.8% (w/w) dextran (500 kDa, Sigma) were prepared in the culture medium. The surface of each well in a 12-well plate was coated with 1 mL of 2% agarose solution (w/v) to create a non-adhesive surface, and after the agarose solidified, all wells were washed with PBS. Subsequently, 1 mL of the 5.0% (w/w) PEG solution was added to each well. Cells, suspended in 10 µL of 12.8% (w/w) aqueous dextran solution at a concentration of 1.5 × 10^6^ cells per well, were then introduced into the wells. Due to the immiscibility of PEG and dextran, the cells accumulated at the interface within 24 h, forming spheroids. The formed spheroids were treated with 15, 30, 60, and 120 nM concentrations of HNPs. After a 7-day treatment period, the structural integrity of the spheroids was examined under a microscope, and the spheroid diameters were analyzed using ImageJ software [27].

#### SERS-based Raman imaging

To observe the 4-ATP molecules in HNPs and demonstrate their therapeutic potential through cell-based SERS imaging, cells were treated with the developed nanoparticles for 4 h. After incubation, the cells were fixed with 4% PFA. All SERS mapping spectra were collected using a WiTech alpha M + Raman Microscopy System (WiTech alpha M +, Germany). The system was calibrated using a Si wafer with a Raman shift of 521 cm^−1^. Samples were excited with a near-infrared diode laser at a wavelength of 785 nm, under 50 mW laser power, using a 50X objective lens. An integration time of 0.2 s was used for all experiments. The laser spot size was calculated to be 1.2 µm using the following equation. The step size was set to 1.5 µm. Raman images of the cells were obtained by filtering based on the 4-ATP peaks at 1085 and 1585 cm^−1^ [30].

#### Statistical analysis

All experiments were performed with at least three replicates, and the results are expressed as mean ± SEM. Statistical analyses were conducted using GraphPad Prism 8.0. The significance of differences was evaluated using student t-test and one-way ANOVA, followed by Bonferroni's post-hoc test, as recommended by the software. A probability level of p < 0.05 was considered statistically significant. Statistical significance is indicated as follows: *p < 0.05, **p < 0.01, ***p < 0.001, ***p < 0.0001.

## Results

### Highly expressed of eEF2 K in TNBC cells, and association of its elevated expression with reduced survival rate in the patients

Kaplan–Meier analysis was performed to evaluate the prognostic significance of eEF2 K expression in triple-negative breast cancer (TNBC) and basal-like breast cancer subtypes. In this study, survival probabilities were compared between patients with high and low eEF2 K expression, utilizing various survival endpoints, including overall survival (OS) and recurrence-free survival (RFS). The figure demonstrates that high eEF2 K expression is associated with poor patient survival in several subtypes of breast cancer. Specifically, in ER(-), PR(-), HER2(-) patients, those with high eEF2 K expression had a significantly lower OS compared to those with low expression, with a hazard ratio (HR) of 1.9 and a statistically significant log-rank p-value of 0.015. Similarly, for patients with basal-like breast cancer (PAM50 subtype), high eEF2 K expression correlated with reduced OS (HR = 1.84, log-rank p = 0.02), further emphasizing the negative impact of eEF2 K on patient prognosis.

**Fig. 3:**
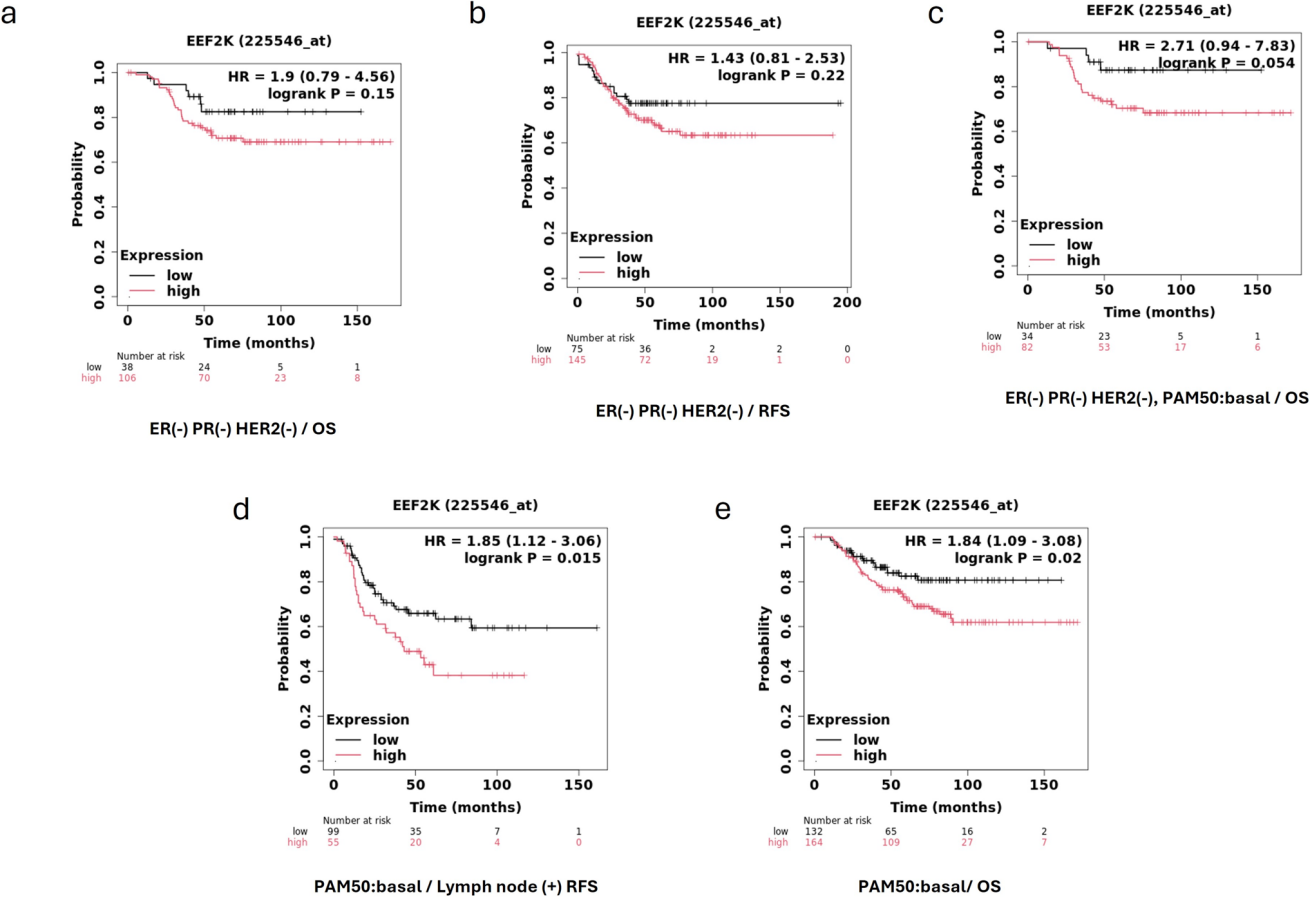
Kaplan–Meier survival analysis of overall survival and recurrence-free survival in breast cancer patients based on eEF2 K expression levels. The survival curves illustrate the differences between patients with high and low eEF2 K expression in various subtypes of breast cancer. (**a**) ER(-), PR(-), HER2(-) overall survival (OS) shows a trend toward poorer outcomes in patients with high eEF2 K expression (HR = 1.9, p = 0.15). (**b**) ER(-), PR(-), HER2(-) RFS indicates statistically with (HR = 1.43, p = 0.22). (**c**) In ER(-), PR(-), HER2(-), PAM50: basal-like subtype, high eEF2 K expression correlates with worse overall survival (HR = 2.71, p = 0.054). (**d**) PAM50: basal subtype overall survival demonstrates a significant reduction in survival for patients with high eEF2 K expression (HR = 1.84, p = 0.02). (**e**) PAM50: basal subtype with lymph node positivity shows a significantly decreased RFS for patients with high eEF2 K expression (HR = 1.85, p = 0.015)

Interestingly, while the relationship between eEF2 K expression and RFS was not statistically significant in ER(-), PR(-), HER2(-) patients (HR = 1.43, log-rank p = 0.22), a notable trend was observed. In the basal-like, lymph-node positive group (PAM50), high eEF2 K expression was significantly associated with reduced RFS (HR = 1.85, log-rank p = 0.015), suggesting that eEF2 K may play a role in promoting tumor recurrence in more aggressive forms of breast cancer. Overall, these findings highlight eEF2 K as a potential biomarker for poor prognosis in TNBC and basal-like breast cancer, particularly in terms of OS and RFS. The differences in hazard ratios and log-rank p-values across different patient subgroups suggest that the impact of eEF2 K on survival may vary depending on the specific breast cancer subtype and other clinical factors. Finally, Kaplan–Meier survival analysis and in vitro experiments consistently demonstrate the therapeutic potential of targeting eEF2 K in TNBC. The elevated eEF2 K expression correlates with worse survival outcomes in specific breast cancer subtypes, and its silencing, as part of an HNP-based therapy, may offer an innovative approach for improving prognosis in TNBC patients Fig. 3.

### Hybrid nanoparticles leading by LbL method as a modular platform for codelivery of eEF2 K-siRNA and quercetin

**Fig. 4:**
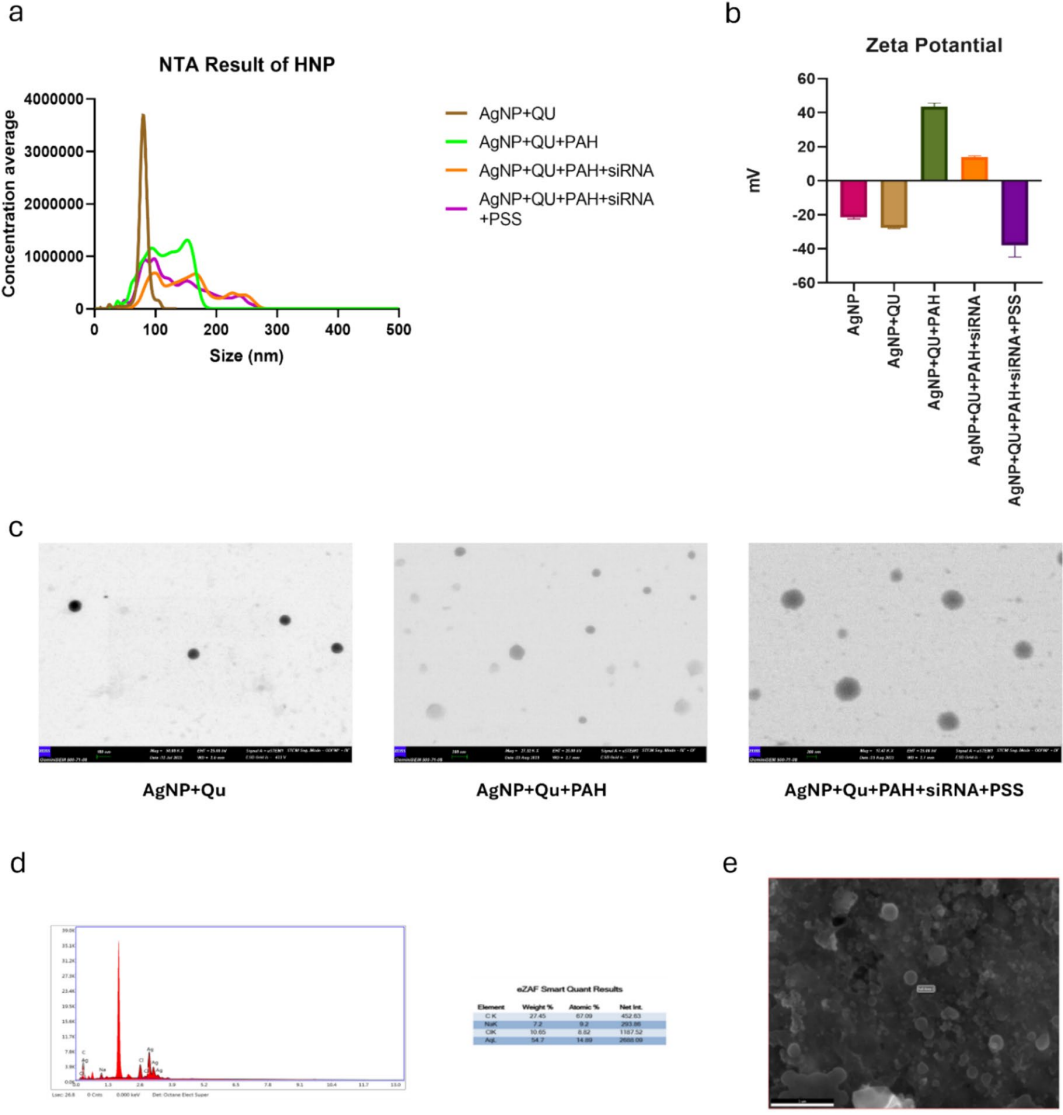
Characterization of HNP. (**a**) Size change of HNP after each layer formation. (**b**) Zeta-potential levels after each electrostatic interaction. (**c**) TEM images for different layers of HNP (200 nm, scale bar). (**d**) Elemental analysis data for HNP from EDX. (**e**) SEM result for HNP (1 µm, scale bar)

As detailed in the methods section, the hybrid nanoparticles developed using the Layer-by-Layer technique were characterized using NTA, ZetaSizer, STEM, SEM and EDX (Fig. 4). The size analysis of HNPs, which increased with each layer added using the LbL method, was conducted using NTA. The AgNP + QU particles, initially 79.5 nm, increased to 117 nm after PAH coating and reached 153 nm upon complexation with eEF2 K-siRNA. After the final PSS coating, the HNPs stabilized at a final size of 133 nm (Fig. 4-a).

The charge difference between each layer, electrostatically bound, was determined by measuring the zeta potentials. The surface charge, which was approximately − 30 mV for AgNP and QU complexes, increased to around + 40 mV after the PAH coating. Upon the addition of eEF2 K-siRNA molecules, the zeta potential decreased to approximately + 18 mV and reached a final value of − 35 mV after the PSS coating (Fig. 4-b).

To understand the morphological structure of the hybrid nanoparticles, imaging was performed using electron microscopy. STEM analysis provided two-dimensional images, allowing for the examination of the dimensional and structural changes in the HNPs. Additionally, to determine whether the particles were spherical or circular, SEM analysis was performed to obtain three-dimensional images. The resulting images confirmed the formation of spherical particles (Fig. 4-c/e) To verify the successful application of the LbL method during particle synthesis, elemental analysis of the particles was conducted. EDX spectra were collected, and the elemental composition of the particles was analyzed by mass percentage. The analysis revealed that the particles contained 54.7% silver and 27.45% carbon by mass (Fig. 4-d).

After the synthesis of the developed HNP particles, the amount of quercetin adsorbed on the AgNP core was determined. For this purpose, a calibration curve was developed by using free quercetin standards at 371 nm wavelength due to the maximum absorbance value of quercetin observed at this point. Following this, the synthesized AgNP + QU complex was centrifuged at 15,000 rpm for 30 min to precipitate the particles, and the supernatant, containing the unbound quercetin molecules, was analyzed by using the calibration curve. Finally, the adsorption efficiency of quercetin molecules was determined as 55.84% (Supplementary Fig. 1).

Similarly, the optimal ratio for complex formation between eEF2 K-siRNA molecules and HNPs was determined. In this context, the concentration of eEF2 K-siRNA was kept constant while the HNP concentrations were varied. The complexes formed at different ratios were analyzed using agarose gel electrophoresis, a method previously established in our laboratory [31]. The most efficient complex was assumed to be 5:1 (NP:siRNA) as a ratio (Supplementary Fig. 2-a).

The zeta potential values of the complexes formed at different ratios of HNP, and eEF2 K-siRNA were also examined to gain insights into the binding ratios. Free siRNA molecules had a zeta potential of − 27 mV, and with increasing HNP concentrations, the surface charge of the particles increased. The most efficient ratio, 5:1 (NP:siRNA), which was the first ratio with a positive surface charge, was measured to be approximately + 18 mV (Supplementary Fig. 2-b).

### Conveniently cellular uptake of HNPs without any external stimuli

In this context, cell nuclei were stained with DAPI to allow fluorescent imaging and the eEF2 K-siRNA molecules used in the HNPs were replaced with FAM-labeled anti-GAPDH-siRNA (FAM-siRNA), allowing the particles to be tracked under the fluorescent microscope due to the FAM tag. The cell nuclei appeared as blue fluorescence from DAPI staining, while the HNPs were visualized as red fluorescence due to the FAM-siRNA. The merged image was obtained by overlaying these two fluorescence images, providing insights into the intracellular localization of the HNPs.

**Fig. 5:**
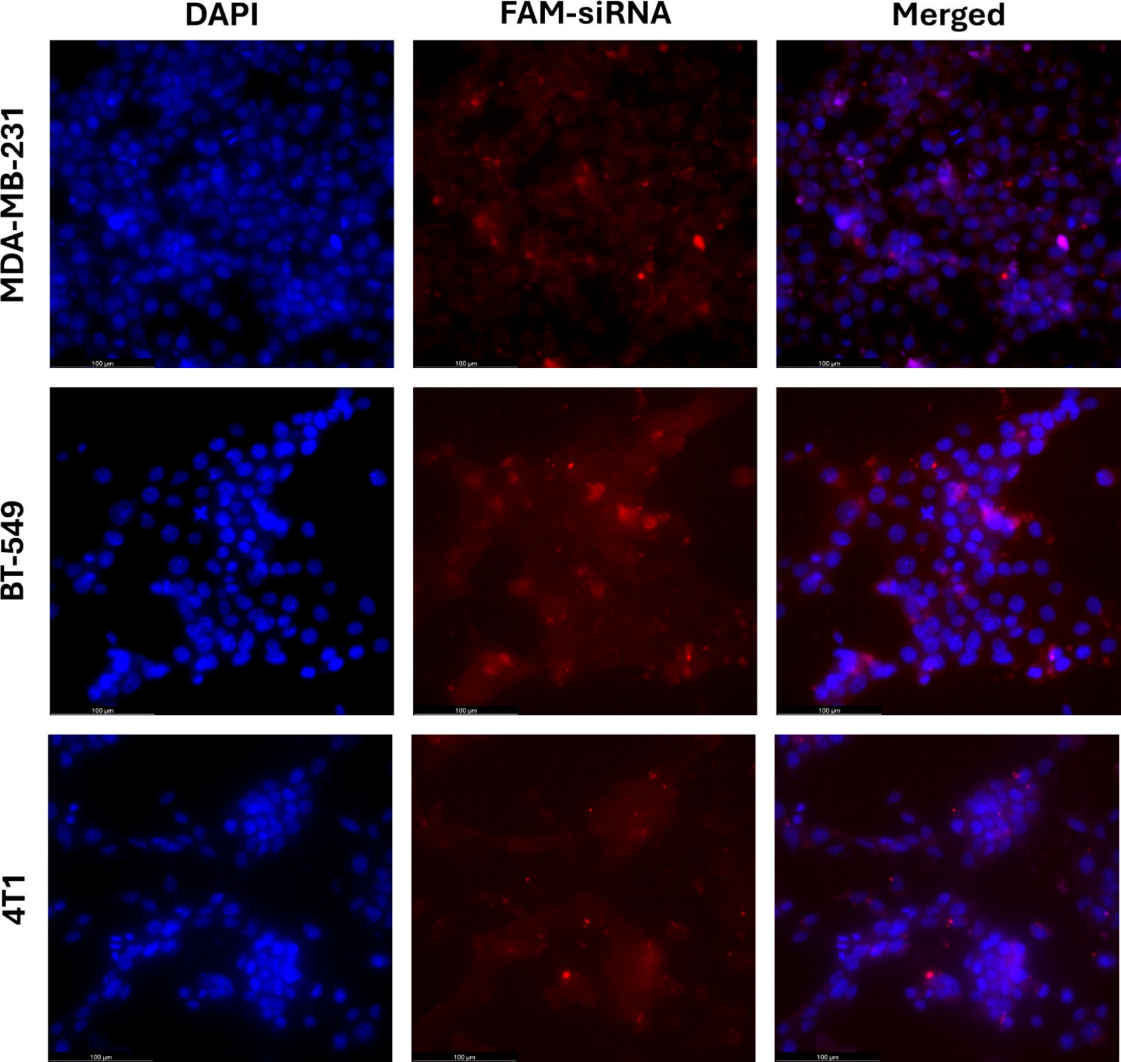
In vitro cellular internalization of HNP for MDA-MB- 231, BT- 549 & 4T1 cells. Fluorescence imaging of cells incubated with hybrid nanoparticles loaded with FAM-labeled anti-GAPDH siRNA for 6 h. Nuclei are stained with DAPI (blue), and the FAM-labeled siRNA (red) indicates successful nanoparticle uptake. Cells were fixed with 4% paraformaldehyde and imaged using a Leica & Dm Il Led Fluo fluorescent inverted microscope (100 µm, scale bar)

As seen in Fig. 5, the merged images show that the red fluorescence from the particles is located adjacent to the blue fluorescence from the cell nuclei in all three cell lines (MDA-MB- 231, BT- 549, and 4T1). This indicates that the particles were successfully internalized by the cells, and cellular uptake did not vary between different cell types/lines. Furthermore, the absence of red fluorescence in the dark areas of the merged images confirms that no particles remained outside the cells and that all delivered particles were readily internalized. This suggests that the designed system does not require any additional stimuli or external forces for cell entry.

### Delivery of eEF2 K-siRNA supporting with quercetin in HNP as potential TNBC therapeutics

**Fig. 6:**
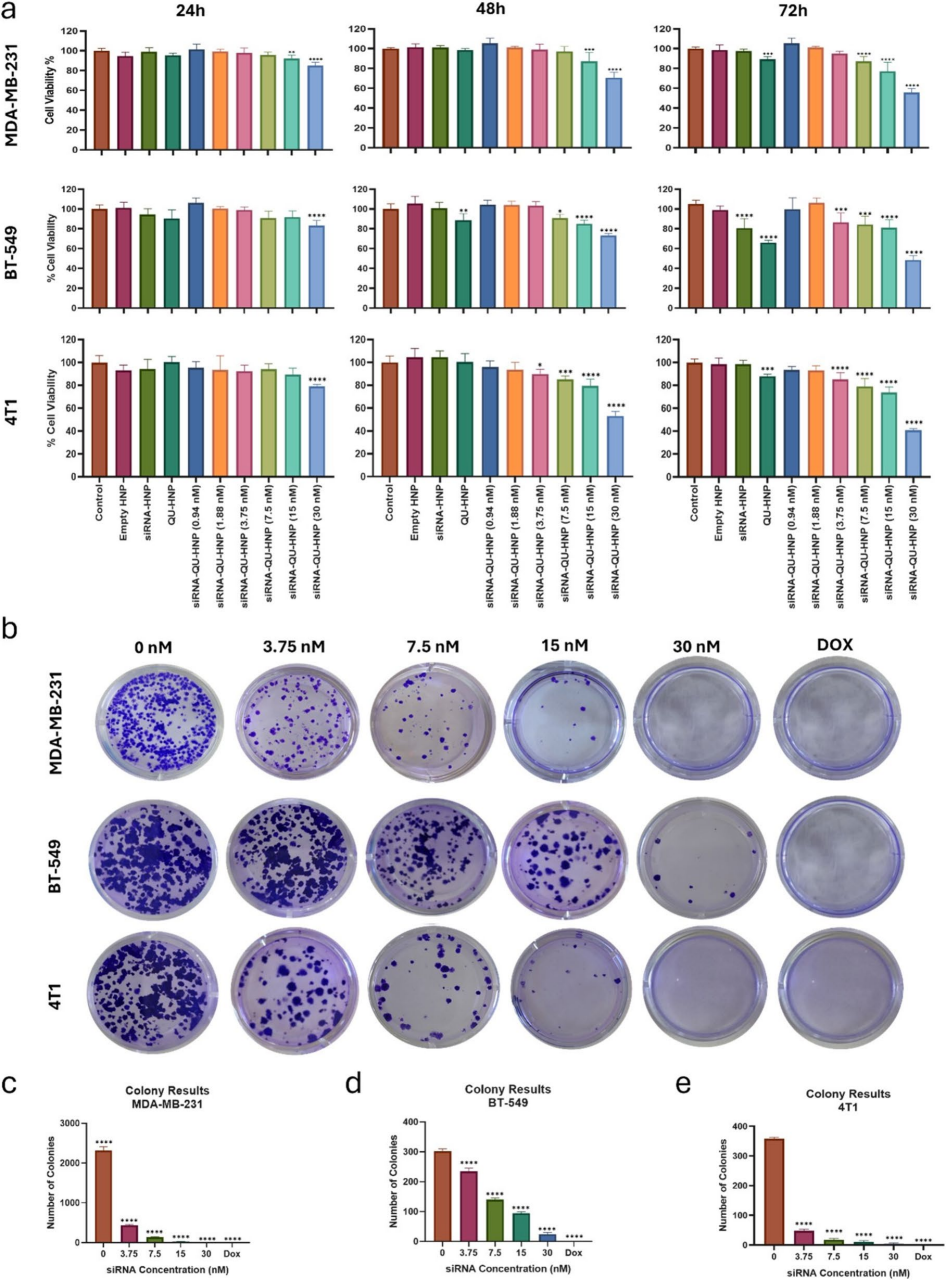
In vitro cellular treatment results. (**a**) Resazurin assay results of empty HNP, only eEF2 K-siRNA loaded HNP (siRNA-HNP), only Quercetin loaded HNP (QU-HNP) and different concentrations of eEF2 K-siRNA & quercetin loaded HNP (siRNA-QU-HNP) for MDA-MB- 231, BT- 549 & 4T1 cells. (**b**) Colony formation assay images for MDA-MB- 231, BT- 549 & 4T1 cells treated with different concentrations of HNP and Doxorubicin (DOX) as a positive control. (**c**-**e**) Quantitative results for colony formation assay retrieved from ImageJ program (in this case, more than 50 cells were considered as a colony). (*: *p* < 0.05, **: *p* < 0.01, ***: *p* < 0.001, ****: *p* < 0.0001; one-way ANOVA calculated in GraphPad program)

The effects of HNPs at concentrations ranging from 0.94 to 30 nM on cell viability were observed in MDA-MB- 231, BT- 549, and 4T1 cell lines over 24, 48, and 72 h. The results revealed a consistent trend across all three cell lines. After 72 h of treatment, 30 nM HNPs reduced cell viability by approximately 50% in all cell lines, while empty nanoparticles lacking eEF2 K-siRNA and quercetin did not affect cell viability. In this way, we concluded that the designed HNP has no toxic effect on the cells, and all of the results from a decrease in cellular viability arose therapeutic effects rather than toxicity. Moreover, as incubation time increased, HNPs containing only quercetin significantly decreased TNBC cell viability. However, they contain only eEF2 K-siRNA, which showed a significant effect on cell viability only in the BT- 549 cell line after 72 h. These results indicate that increased HNP concentration and treatment duration reduced cell viability across all three TNBC cell lines (Fig. 6-a).

Then, we simulated an environment covering a small amount of cancer cells to understand how HNP treatment effects on the colony formation of the cancer cells. In this way, we expected to observe the capacity of HNPs to suppress both the initial formation of cancerous tissue from a cancer cell, and the potential relapse of the disease following treatment. As shown in Fig. 6b, colony formation was analyzed in three TNBC cell lines over a 13-day period. In untreated control wells (0 nM), cancer cells successfully proliferated, forming colonies that covered the entire well surface by the end of the incubation period. However, treatment with 30 nM HNPs completely inhibited colony formation, demonstrating the strong anti-proliferative effect of HNPs. Doxorubicin (DOX), a well-known chemotherapy drug, was used as a positive control, further validating the effectiveness of the HNP treatment.

The ability of HNPs to inhibit colony formation highlights their potential in preventing both the growth of cancerous tissue from individual cancer cells and the recurrence of cancer post-treatment. This assay provided valuable insights into the long-term therapeutic potential of HNPs and their impact on limiting the tumorigenic capacity of cancer cells (Fig. 6-c/d/e).

### Slowing down the migration of TNBC type cell lines after HNP treatment

In this study, we employed a wound healing assay to evaluate the impact of HNPs on the migratory capacity of TNBC cells. Specifically, the assay was performed using three TNBC cell lines (MDA-MB- 231, BT- 549, and 4T1) to assess the effect of different concentrations of HNPs (ranging from 0.94 to 30 nM) on cell migration over a 72-h period.

**Fig. 7:**
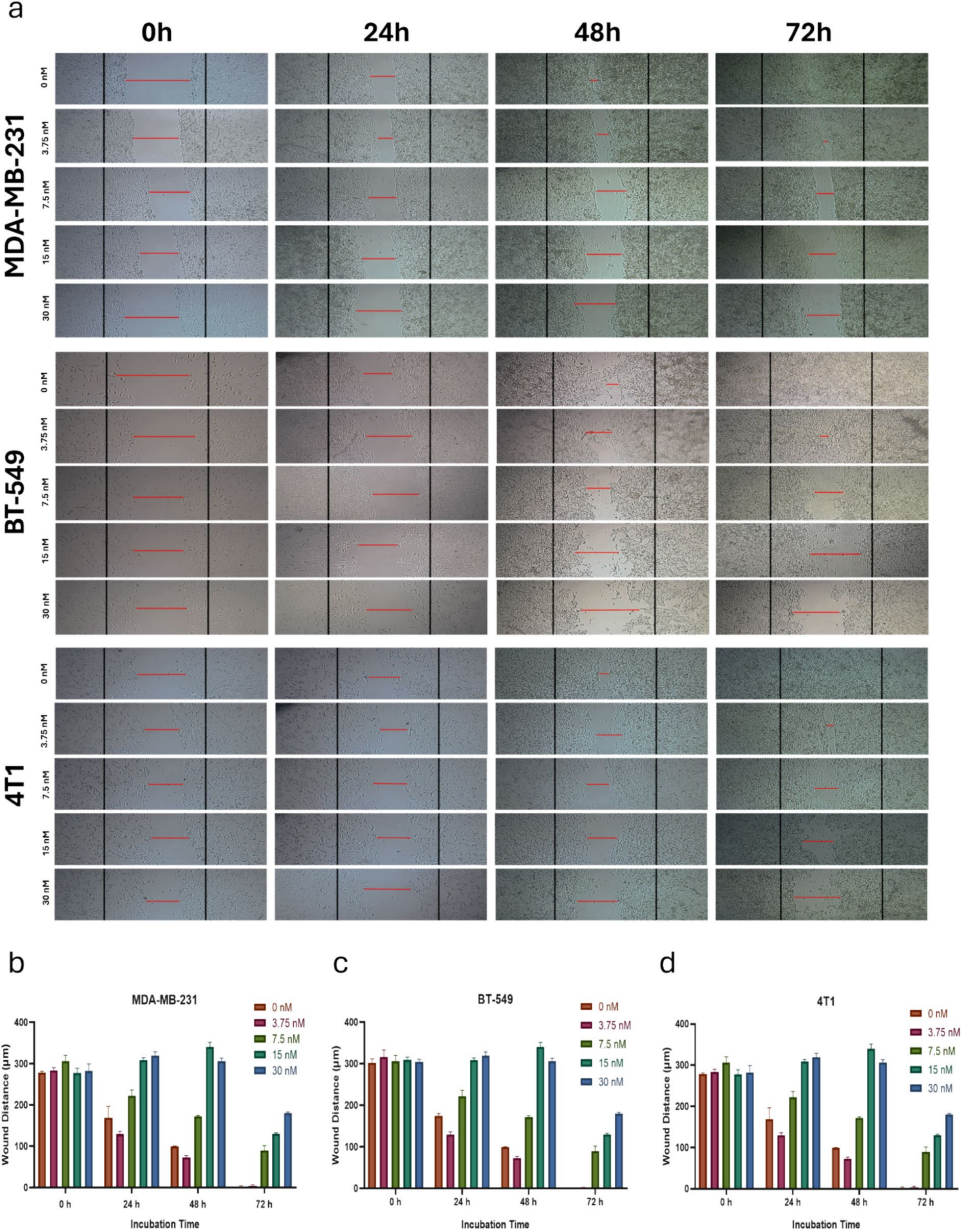
(**a**) In vitro*,* wound healing (migration assay images for MDA-MB- 231, BT- 549 & 4T1 cells treated with different concentrations of HNP. (**b**-**d**) Quantitative results for wound distance in µm scale calculated with ImageJ program and analyzed with one-way ANOVA in GraphPad (*: *p* < 0.05, **: *p* < 0.01, ***: *p* < 0.001, ****: *p* < 0.0001)

As shown in Fig. 7, untreated cells (0 nM HNPs) fully closed the wound within 72 h, indicating active cell migration. In contrast, cells treated with increasing concentrations of HNPs demonstrated significant delays in wound closure, with the highest dose of 30 nM HNP exhibiting the most pronounced inhibitory effect. Microscopic analysis (Fig. 7-a) revealed that, at this concentration, wound closure was largely hindered, suggesting that the nanoparticles significantly.

impaired the migratory capacity of the cancer cells. This inhibition of migration was consistent with the trends observed in our previous cell viability and colony formation assays, where the 30 nM HNP treatment led to a substantial reduction in both cell survival and proliferation.

The results from this assay suggest that HNPs may suppress cell migration in a dose-dependent manner, which is a crucial factor in limiting TNBC metastasis. In TNBC, overexpression of eEF2 K has been shown to enhance cell migration and invasion by activating oncogenic pathways such as Src, FAK, PI3 K/Akt, and c-Myc, as well as promoting epithelial-to-mesenchymal transition (EMT) through the integrin β1/Src/FAK axis and cyclin D1 signaling. These pathways are key drivers of tumor progression, drug resistance, and metastatic spread [32–34]. Notably, knockdown of eEF2 K has been demonstrated to significantly inhibit cell motility by suppressing these signaling cascades. Our findings align with these reports, as we observed a concentration-dependent inhibition of migration upon treatment with eEF2 K-siRNA and Quercetin-loaded HNPs. This suggests that HNPs may attenuate TNBC metastasis by downregulating eEF2 K and subsequently impairing the activity of migration-associated pathways. As illustrated in Fig. 7, although complete inhibition of migration was not achieved, the observed delay in wound closure indicates that HNP treatment may significantly slow the metastatic potential of TNBC cells, providing a promising therapeutic strategy to mitigate tumor progression.

### Necrotic activation of HNP on TNBC cell lines

The apoptosis assay, utilizing Annexin V and PI staining, is employed to determine whether treated cells undergo apoptosis or necrosis. Annexin V binds to phosphatidylserine, a phospholipid typically located on the inner side of the cell membrane. During apoptosis, the cell membrane flips, exposing phosphatidylserine to the cell's exterior, allowing Annexin V to bind and signal the occurrence of apoptosis. On the other hand, PI (propidium iodide) stains nucleic acids like DNA and RNA. In cells undergoing apoptosis or necrosis, membrane permeability increases, allowing PI to enter and bind to DNA, resulting in fluorescence. In healthy cells with intact membranes, PI is excluded due to its size, preventing the generation of a signal. This principle is utilized for analysis through flow cytometry [35].

**Fig. 8:**
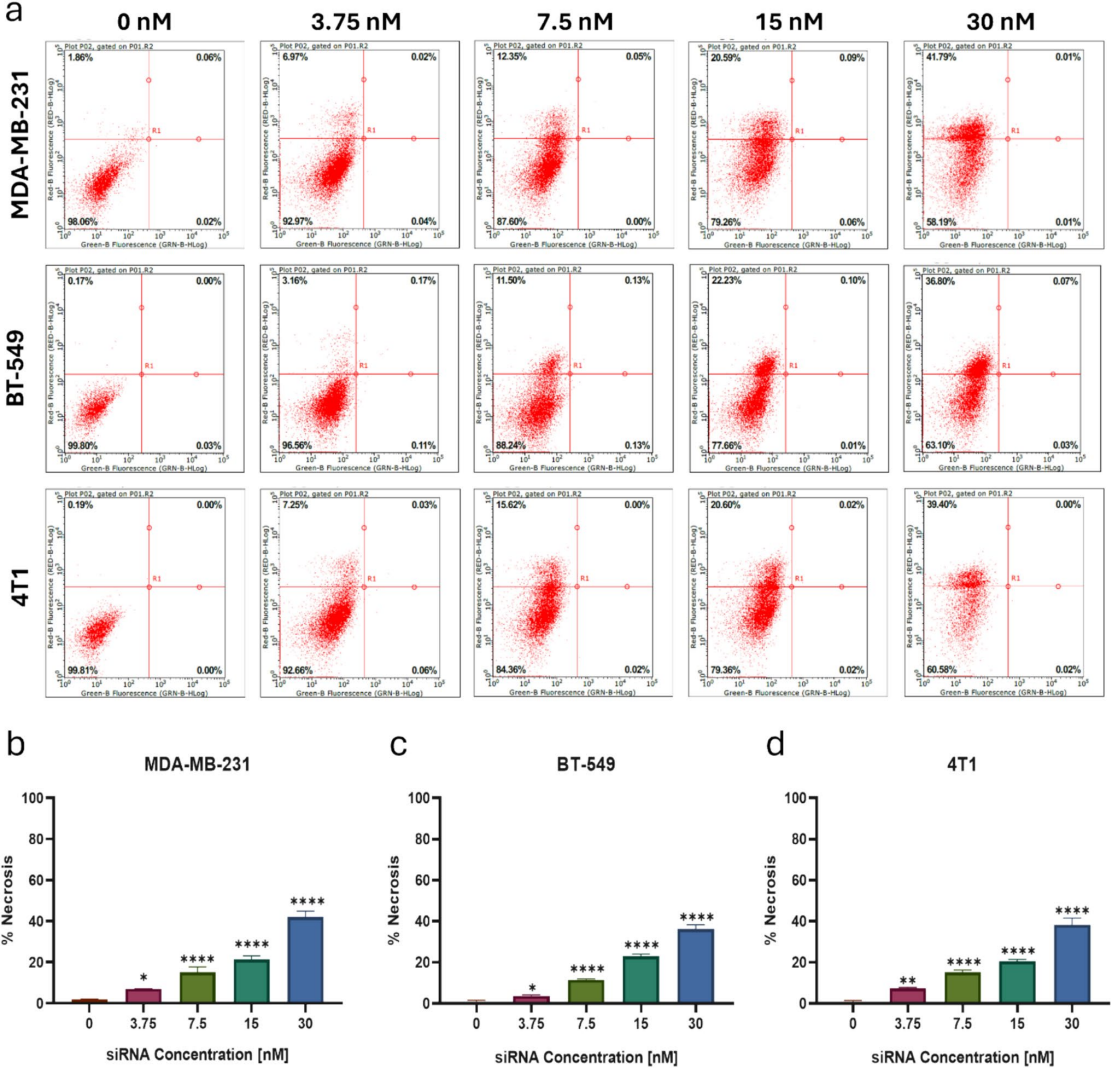
(**a**) Apoptosis assay (Annexin V & PI staining) results for MDA-MB- 231, BT- 549 & 4T1 cells treated with different concentrations of HNP. Quantitative results of necrotic levels of (**b**) MDA-MB- 231, (**c**) BT- 549 & (**d**) 4T1 cells, respectively. (*: *p* < 0.05, **: *p* < 0.01, ***: *p* < 0.001, ****: *p* < 0.0001; one-way ANOVA calculated in GraphPad program)

Following a 72-h HNP treatment at different concentrations ranging from 0 to 30 nM, cells were stained using the mentioned method and analyzed via flow cytometry. The results, presented in Fig. 8-a, categorize cells into four quadrants: the lower left represents healthy cells, the lower right shows early apoptotic cells, the upper right indicates late apoptosis, and the upper left reflects necrotic cells. Gating was performed based on unstained, Annexin V-only, and PI-only controls to establish appropriate fluorescence compensation and minimize spectral overlap. A dot plot of Annexin V-FITC (x-axis) versus PI (y-axis) was generated, and distinct quadrants were used for classification: viable cells (Annexin V-/PI-), early apoptotic cells (Annexin V +/PI-), late apoptotic cells (Annexin V +/PI +), and necrotic cells (Annexin V-/PI +). This approach ensured accurate discrimination between apoptosis and necrosis while preventing misclassification due to late-stage apoptosis or secondary necrosis.

As shown in the bar graphs illustrated in Fig. 8-b/c/d, a notable increase in the percentage of necrotic cells was observed with higher treatment concentrations. In particular, the highest dose of 30 nM HNP significantly elevated the necrosis rate across all three cell lines, resulting in approximately 35% to 40% necrotic cells. These findings indicate that the 30 nM dose is the most effective concentration, exerting a strong therapeutic effect on the cancer cells in all three TNBC lines. Furthermore, this dose significantly increased the permeability of the cell membrane, leading to cell death. A similar trend in response to treatment was observed in the MDA-MB- 231, BT- 549, and 4T1 cell lines.

Notably, the MDA-MB- 231 cell line exhibited a higher rate of necrosis compared to the other two cell lines, highlighting its greater susceptibility to HNP treatment. Breast cancer cell lines exhibit distinct biological and genetic properties. The MDA-MB- 231 cell line, a metastatic TNBC model, is derived from human breast invasive ductal carcinoma and is highly aggressive. It is characterized by the absence of estrogen receptor (ER-), progesterone receptor (PR-), and human epidermal growth factor receptor 2 (HER2-), making it a representative model for studying invasive TNBC [36–38]. In contrast, the BT- 549 cell line, classified as a primary TNBC model, originates from human papillary invasive ductal carcinoma and also lacks ER, PR, and HER2 expressions. However, BT- 549 cells harbor mutations in PTEN, RB1, and TP53, whereas MDA-MB- 231 cells carry mutations in BRAF, CDKN2 A, KRAS, NF2, and TP53, contributing to differences in tumorigenicity and invasiveness [37–39]. Given that TNBC subtypes respond differently to therapeutic agents [40, 41], we hypothesize that the higher necrosis rates observed in MDA-MB- 231 cells compared to other cell lines may be attributed to their enhanced metastatic potential and unique genetic alterations that influence cell death pathways.

### Analysis of intracellular protein level changes and understanding of necroptosis and parthanatos mechanisms of cell death activation with HNP treatment

**Fig. 9:**
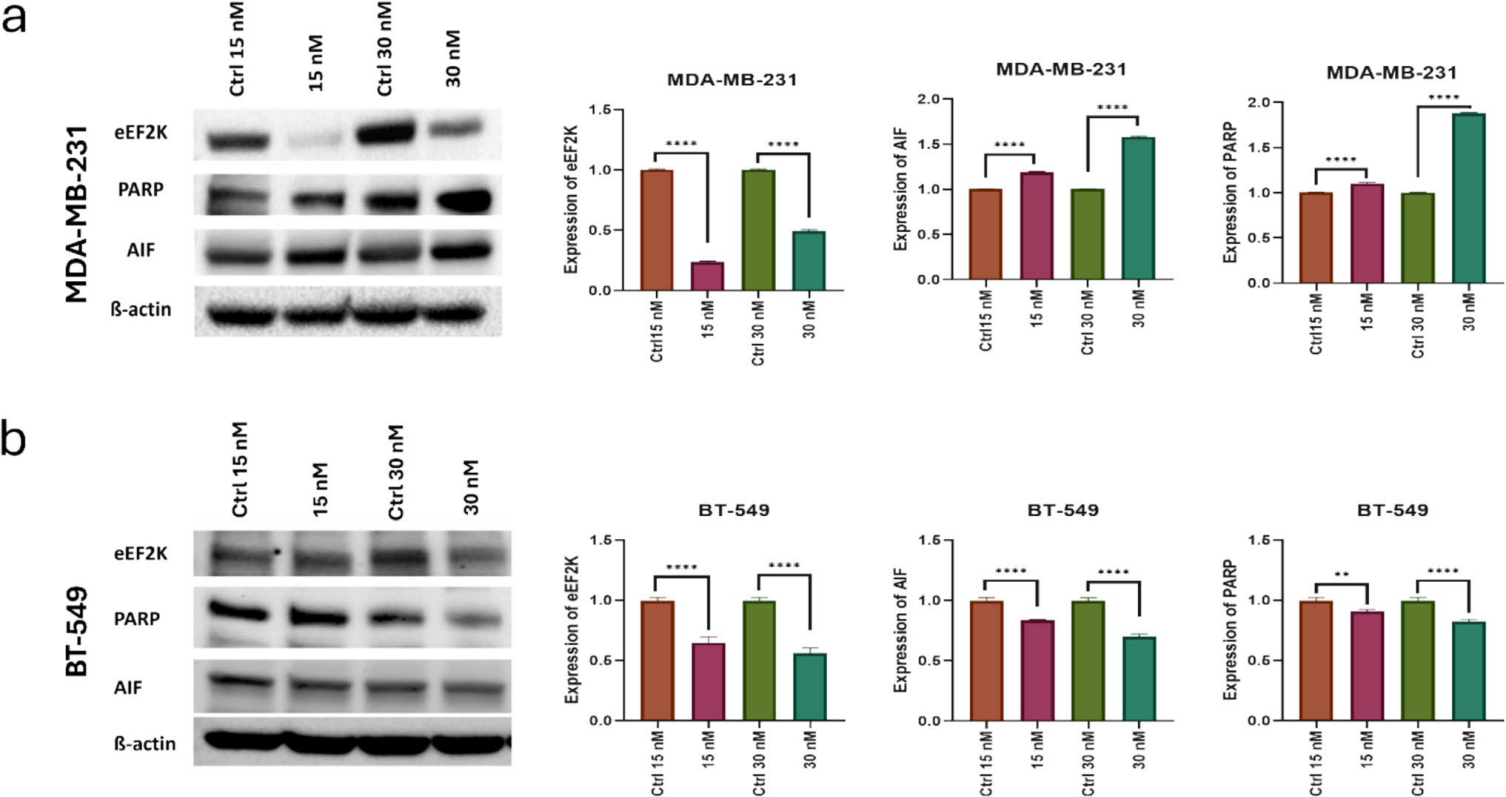
Western blot assay results for (**a**) MDA-MB- 231 & (**b**) BT- 549 cell lines treated with higher concentrations of HNPs and their control groups which is empty nanoparticles in the same concentration. (*: *p* < 0.05, **: *p* < 0.01, ***: *p* < 0.001, ****: *p* < 0.0001; student t-test calculated in GraphPad program)

In this study, intracellular protein level changes following the treatment with hybrid nanoparticles were analyzed using Western blot. We found that HNP treatment led to marked inhibition of eEF2 K expression in both MDA-MB- 231 cells and BT- 549 cells (Fig. 9). In addition to we found that HNP treatment significantly expressions of poly [ADP-ribose] polymerase 1 (PARP- 1) and apoptosis inducing factor (AIF) in MDA-MB- 231 cells, while suppressed PARP- 1 and AIF in BT- 549 cells compared to control cells (Fig. 9).

For anti-cancer therapy, the successful induction of cell death of the tumor is one of the most important objectives. Recent studies are reported that overexpression of PARP1 can promote necroptosis (a regulated form of cell necrosis). Regulated necrosis includes a wide variety of cell death pathways that share characteristic of necrotic processes. One of these pathways is PARP- 1-mediated cell death, which is named as parthanatos [42].

Under pathological conditions, PARP- 1 over-activation causes the accumulation of PAR polymers and nuclear translocation of AIF, where it causes chromatin condensation and DNA fragmentation. Therefore, AIF is a key mediator in parthanatos, which is independent of caspase activation [43–45]. Our results showed that mechanisms for the cell death of the two cells were different. Necrotic Cell death mechanisms varied between the cell lines, as MDA-MB- 231 were found to die by PARP overexpression, while BT- 549 cells by suppression of PARP expression, likely due to the differences in genetic background. When the overall results are evaluated, it is evident that our gene therapy agent worked effectively in all cell lines, successfully reducing eEF2 K protein levels. Moreover, our results strongly suggest that HNP treatment is mediated PARP- 1-dependent mechanism of cell death in the human tumor cell lines MDA-MB- 231.

### Inhibiting spheroid formation of TNBC cell lines with HNP treatment

The 3D spheroid assay is a necessary tool for mimicking the in vivo environment in cancer research. Unlike traditional 2D cell cultures, which grow cells in a monolayer, the 3D spheroid model enables the formation of spherical clusters of cells that closely resemble the architecture of solid tumors. This three-dimensional structure allows us to evaluate drug penetration, cell interactions, and the overall behavior of tumor cells within a more realistic tumor microenvironment. Tumor tissues in the body have layers of cells with varying access to nutrients and oxygen, leading to differences in treatment response. The spheroid assay helps replicate these conditions, making it a valuable model for preclinical studies of cancer therapeutics.

In our study, we used the 3D spheroid assay to understand how our hybrid nanoparticle treatment affects tumor-like structures compared to traditional 2D models. In 2D experiments such as cell viability, colony formation, and migration assays, cells are plated in a monolayer, allowing for direct contact with the treatment, leading to fast and effective outcomes. However, in real-life tumor scenarios, therapeutic agents primarily interact with cells at the tumor's surface, with limited penetration into deeper layers. This challenge is replicated in the 3D spheroid model, making it essential for evaluating how well a treatment penetrates and impacts the entire tumor mass.

**Fig. 10:**
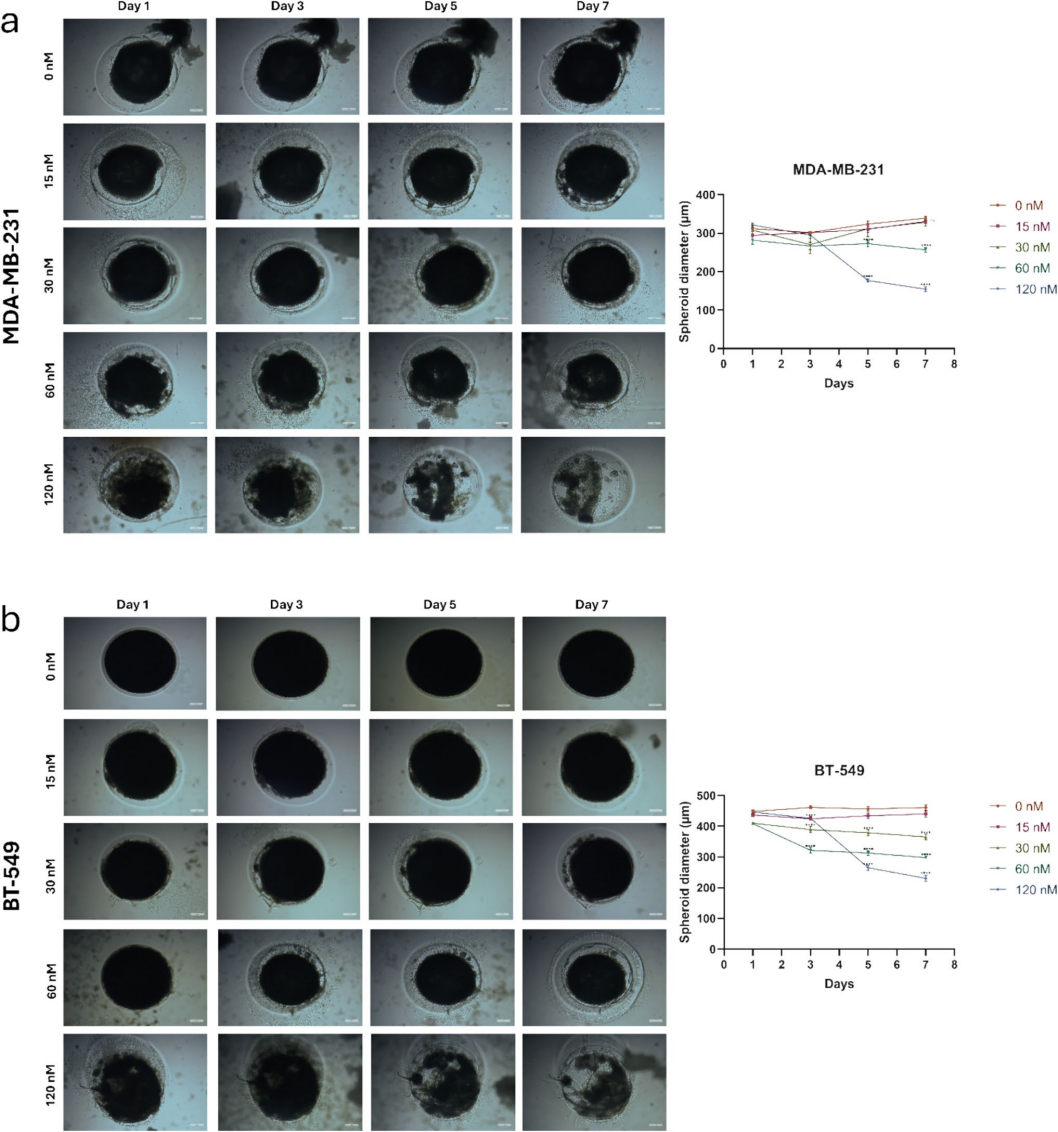

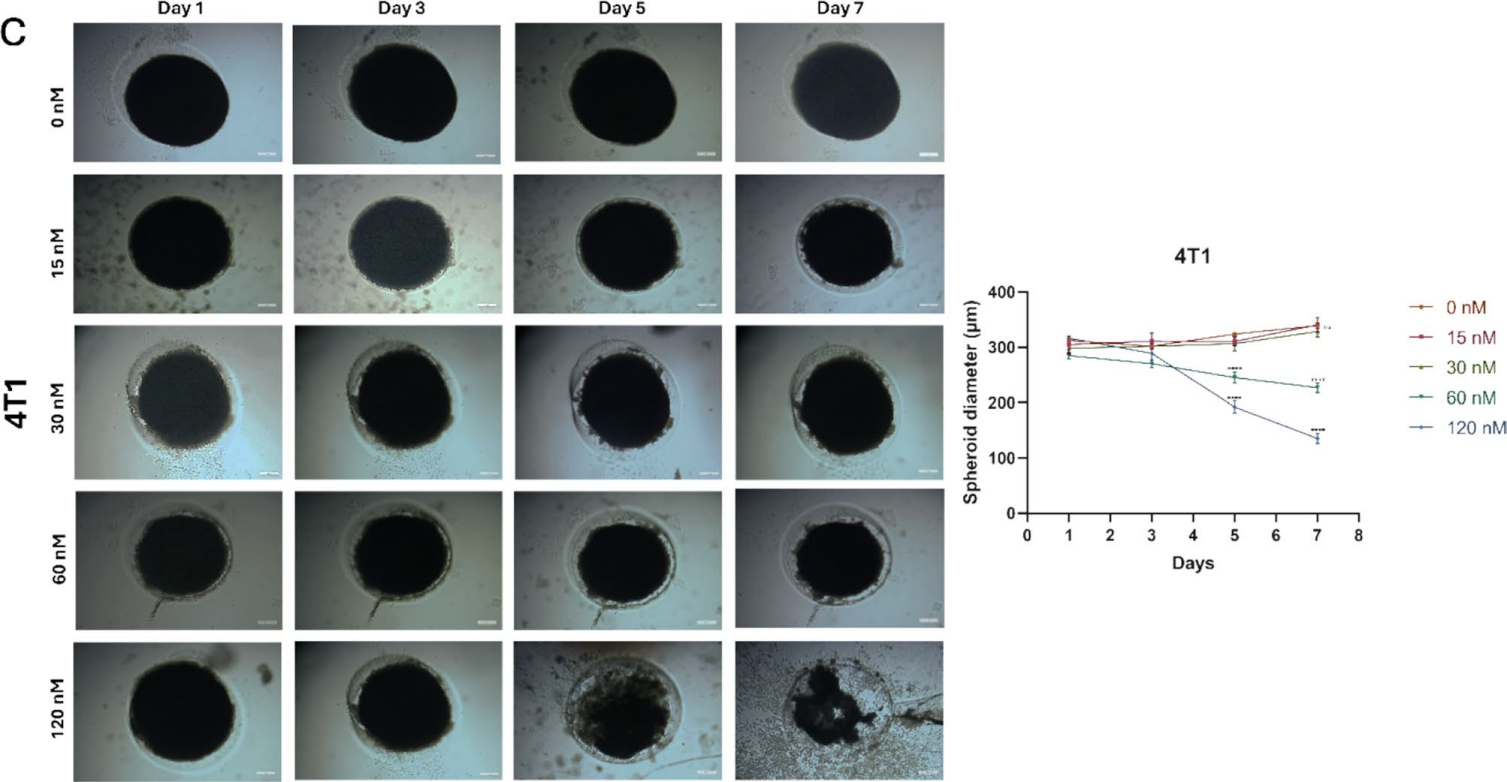
Spheroid formation and treatment with HNPs. Spheroids images formed at the PEG-dextran interface 24 h post-seeding of 1,500,000 cells/well. They were treated with 15, 30, 60, and 120 nM concentrations of HNPs for 7 days and obtained quantitative results of spheroid diameters of (**a**) MDA-MB- 231, (**b**) BT- 549 & (**c**) 4T1 cells, respectively. (*: *p* < 0.05, **: *p* < 0.01, ***: *p* < 0.001, ****: *p* < 0.0001; one-way ANOVA calculated in GraphPad program) (5 µm, scale bar)

In the 3D spheroid assay, we used TNBC (triple-negative breast cancer) cell lines to grow multicellular spheroids and tested different concentrations of HNP treatment. While the concentrations used in 2D experiments were sufficient, they proved inadequate for 3D spheroids due to their more complex and layered structure. Therefore, we increased the HNP doses to 60 nM and 120 nM and extended the treatment period to seven days to observe long-term effects on the spheroids (Fig. 10).

The results of our experiment, shown in Fig. 10, demonstrate that the 120 nM HNP treatment reduced spheroid diameters by half after seven days, indicating significant tumor shrinkage. The 60 nM dose also showed a slight effect, though not as pronounced, while the other doses did not produce any statistically significant changes. This indicates that a higher dose of HNP is required to effectively penetrate and reduce the size of the spheroids, which more accurately represents solid tumors.

These findings underline the importance of the 3D spheroid model in preclinical studies, as it highlights the challenges of drug delivery in a more complex tumor environment. It also shows the necessity of optimizing treatment concentrations and durations for effective tumor control in vivo. By using the 3D spheroid model, we were able to better understand the potential of HNP as a therapeutic agent for treating TNBC, providing a more accurate evaluation than could be achieved with 2D cell cultures alone. Lastly, the success of our HNP treatment in this assay highlights its potential for overcoming the challenges of drug delivery in solid tumors and opens the door for further in vivo testing.

### Tracking of HNPs under Raman spectroscopy by gaining theranostic properties

**Fig. 11:**
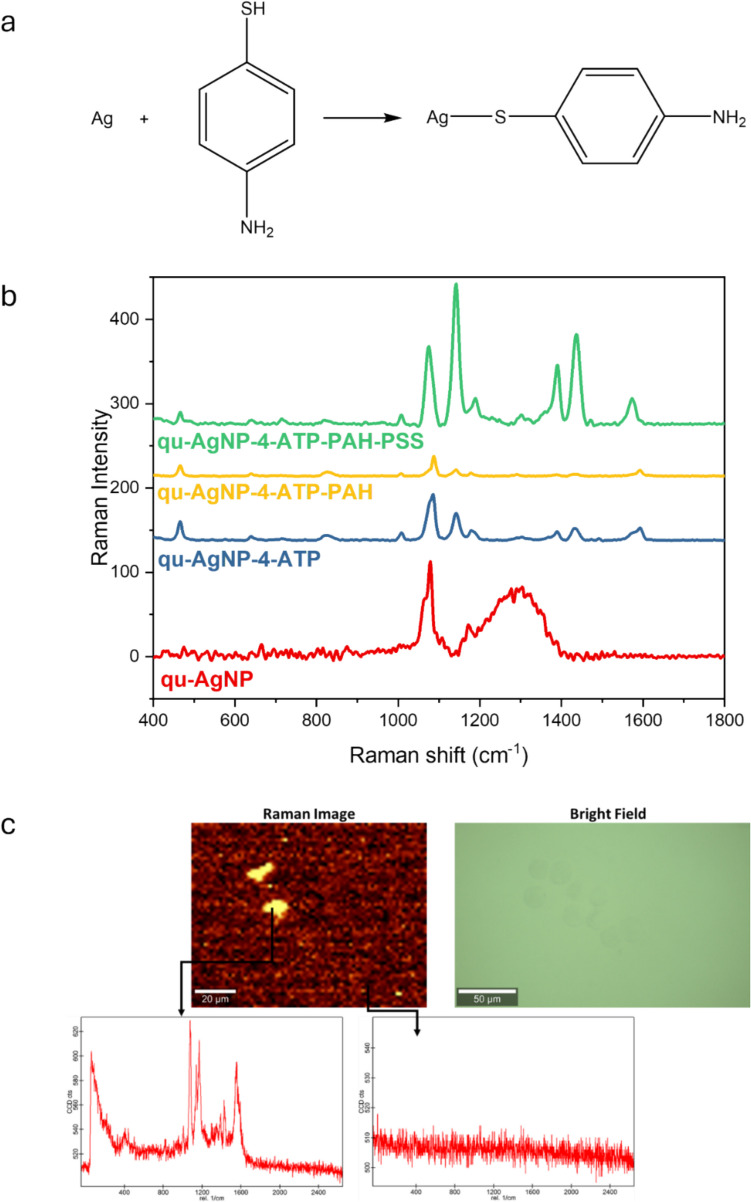
Theranostic properties of HNP under Raman spectroscopy results. (**a**) Synthesis illustration for 4-ATP conjugation of HNP. (**b**) Raman spectra taken from each layer of the HNP structure. (**c**) The images of HNPs administered to the cells under Raman spectroscopy and the spectra obtained from these images (20 µm & 50 µm scale bar for Raman image and Bright field, respectively)

After all cellular experiments were performed successfully, we just wondered whether the hybrid nanoparticles gained the ability to be tracked based on their location under Raman spectroscopy. In this purpose, thanks to the Raman-active molecule 4-ATP (4-Aminothiophenol), we produced.

a theranostic nanoparticles with HNPs. Through the synthesis process, a sulfur bond was formed between the silver atoms and the -SH group of the 4-ATP molecules, allowing the system to be modified (Fig. 11-a).

In the SERS spectra of the synthesized theranostic nanoparticles, peaks corresponding to C–C bonds at 1590 cm^−1^ and C-S bonds at 1086 cm^−1^ from 4-ATP were observed. These peaks, along with the shifts they exhibit, were used to characterize the theranostic nanoparticles, and their cellular uptake was subsequently examined. After the conjugation of 4-ATP, the nanoparticles were coated with the polymers PAH (Polyallylamine Hydrochloride) and PSS (Polystyrene Sulfonate). It was observed that the 4-ATP peak at 1086 cm^−1^ shifted to 1075 cm^−1^, and the peak at 1590 cm^−1^ shifted to 1576 cm^−1^, further confirming the characterization of the nanoparticles (Fig. 11-b).

Due to the 4-ATP conjugation, the destination of HNP carriers within the body, as well as the tissues and organs where they accumulate, can be tracked using Raman spectroscopy. Based on the spectra and images obtained from the cells under Raman, it can be easily concluded that the designed particles have acquired theranostic properties (Fig. 11-c).

## Discussion

The present study introduces a novel HNP system developed using the LbL method for the co-delivery of eEF2 K-siRNA and quercetin, aimed at treating TNBC. Our results demonstrate that this modular platform not only efficiently delivers these therapeutic agents but also offers significant advantages in terms of cellular uptake, anti-tumor activity, and the ability to track nanoparticles under Raman spectroscopy.

One of the most significant aspects of our work is the modularity of the LbL technique. By sequentially depositing positively and negatively charged polymers (PAH and PSS, respectively) we successfully loaded eEF2 K-siRNA and quercetin in distinct layers of the nanoparticles. This method allowed us to evaluate the synergistic effects of siRNA and quercetin both individually and in combination, confirming that the dual-therapy approach has a more substantial impact on TNBC cells than either agent alone. This modular design opens the door to further customization of nanoparticle-based therapies, offering a flexible platform that can be adapted for other therapeutic targets beyond TNBC.

Our results showed that the HNPs were taken up by TNBC cells efficiently without the need for external stimuli. This property is crucial for in vivo applications, where external intervention may not be feasible. The electrostatic interactions between the nanoparticle surface and the negatively charged cell membrane likely facilitated this uptake. The rapid internalization of the nanoparticles ensures that the therapeutic agents reach their intracellular targets, such as eEF2 K, in a timely and effective manner.

The codelivery of eEF2 K-siRNA and quercetin in the HNP platform significantly suppressed the viability of TNBC cells. This outcome aligns with previous findings that eEF2 K inhibition, coupled with chemotherapeutic agents, can induce apoptosis in cancer cells. The known ability of quercetin to induce oxidative stress and apoptosis in cancer cells likely complements the eEF2 K-siRNA, which silences a key survival pathway in TNBC cells. The combined action of these agents in the HNPs led to a more pronounced reduction in cell viability and colony formation compared to individual treatments.

In vitro migration assays showed that the HNPs significantly inhibited the migratory capacity of TNBC cell lines. Given that metastasis is a major contributor to poor prognosis in TNBC patients, the ability of our HNPs to slow down cell migration holds great potential for reducing metastatic spread. This finding suggests that the dual-action HNPs not only combat the primary tumor but could also help prevent or mitigate the dissemination of cancerous cells.

A notable observation in our study was the necrotic activation of TNBC cells at high siRNA concentrations. Our data suggests that our developed nanodelivery system can induce necrotic pathways in TNBC cells. AgNPs with varying surface modifications can induce different forms of cell death [46]. In a separate study, it was observed that treatments using AgNPs suppressed apoptosis while promoting necrosis [47]. Based on this, it can be inferred that while quercetin and eEF2 K-siRNA molecules aimed to promote apoptosis by increasing the levels of apoptotic pathway proteins, the presence of AgNPs in the system diverted the cells toward necrotic death rather than apoptosis.

The suppression of eEF2 kinase activity has been demonstrated to increase PARP levels in nasopharyngeal carcinoma cells [48, 49]. PARP1 has diverse biological functions such as DNA repair, apoptosis, necrosis, etc. [50], is activated by DNA damage. When DNA is moderately damaged, PARP- 1 participates in the DNA repair process and the cell survives. However, the case of extensive DNA damage overactivation of PARP- 1 leads to necrotic cell death, which is named as parthanatos PARP- 1 inhibitors in cancer chemotherapy [51–53]. Parthanatos is a multistep pathway that plays a pivotal role in tumorigenesis. It was showed that by induced parthanatos inhibited breast cancer cell growth [54]. There are many molecules in the parthanatos cascade that can be exploited to create therapeutic interventions, including PARP1 and AIF. These critical molecules are involved in tumor cell proliferation, progression, invasion, and metastasis. Therefore, these molecular signals in the parthanatos cascade represent promising therapeutic targets for cancer therapy [55]. We demonstrated that in HNP treatment led to necrotic cell death in MDA-MB- 231 cells is mediated by PARP- 1-dependent mechanism of cell death. In addition, HNP treatment induced necrotic cell death in BT- 549 cells by different mechanism. Our results strongly suggest that HNP treatment may be a potential treatment for breast cancer patients.

One of the key challenges in cancer treatment is the ability to target tumor spheroids, which better mimic the 3D structure of in vivo tumors. Our experiments revealed that HNP treatment effectively reduced the size of TNBC spheroids, with 120 nM concentrations showing the most significant reduction in spheroid diameters by the end of seven days. This finding indicates that the nanoparticles penetrate tumor-like structures effectively, making them a promising candidate for future in vivo studies.

Finally, the theranostic potential of our HNPs was confirmed through their traceability under Raman spectroscopy. Incorporating 4-ATP molecules into the AgNP core provided a reliable way of tracking the nanoparticles within cells and tissues. This property is invaluable for monitoring therapeutic distribution and verifying that the nanoparticles reach their intended targets, offering a dual diagnostic and therapeutic function that can enhance treatment precision in clinical settings.

## Conclusion

The development of HNPs in this study introduces an innovative approach to the treatment of TNBC, offering not only therapeutic benefits but also the ability to track cellular interactions through SERS-based detection. The results from in vitro studies demonstrated that this approach is both effective and successful.

Upon examining the results from 3D spheroid experiments, it is clear that our approach's therapeutic impact extends beyond the cellular level to tissue and organ dimensions. Consequently, this suggests that future research could continue with in vivo studies, potentially advancing to clinical phase trials.

Although our HNP system demonstrates promising in vitro efficacy, its in vivo translation presents potential challenges related to biodistribution, immune response, and clearance mechanisms. Systemically administered nanoparticles often encounter rapid clearance by the mononuclear phagocyte system (MPS), limiting their circulation time and tumor accumulation. While the EPR effect may enhance passive tumor targeting, its variability across tumor models necessitates further investigation. Additionally, endosomal entrapment remains a key barrier in siRNA delivery, although our system's positively charged PAH layer may facilitate endosomal escape. The presence of AgNPs as the core also raises biocompatibility and long-term safety considerations, which require further study. Future in vivo pharmacokinetic and biodistribution studies and potential surface modifications such as PEGylation or biomimetic coatings may help optimize the therapeutic potential of HNP-based siRNA therapy for TNBC treatment.

Furthermore, HNP particles, which offer a highly manipulable and efficient therapeutic platform, could be applied to various types of cancers and diseases by using different metallic nanoparticles, flavonoids, and genetic materials. Future studies could also explore the application of this system to combat antibiotic-resistant bacteria or in the genetic treatment of various neurological diseases. Additionally, it is anticipated that the developed HNP system could play a role in the development of mRNA vaccines or cancer vaccines, particularly in the context of a potential pandemic.

## Supplementary Information

Below is the link to the electronic supplementary material.

Supplementary file1 (DOCX 255 KB)

## Data Availability

The data underlying this study is accessible through the corresponding author upon justified request.
