# Supplementary material for: Development of silver-based hybrid nanoparticles loaded with eEF2 K-siRNA and quercetin against triple-negative breast cancer
Source: Drug Deliv Transl Res. 2025 Apr 23;16(1):268–90. doi: 10.1007/s13346-025-01860-6 (PMC12682719; doi:10.1007/s13346-025-01860-6)
Supplement: Supplementary file 1 — Supplementary file1 (DOCX 255 KB) [file 13346_2025_1860_MOESM1_ESM.docx]

**Development of Silver-based Hybrid Nanoparticles Loaded with eEF2K-siRNA and Quercetin Against Triple-Negative Breast Cancer**

*Orhan Burak Eksi^1,2^, Ahsen Guler^3,4^, Munevver Akdeniz^2,5^, Pınar Atalay^6^, Zuhal Hamurcu^3,4^, Omer Aydin ^1,2,5,7*^*

^1^ ERNAM-Nanotechnology Research and Application Center, Erciyes University, Kayseri, 38039, Turkey

^2^ NanoThera Lab, ERFARMA-Drug Application and Research Center, Erciyes University, Kayseri, 38039, Turkey

^3^ Department of Medical Biology, Faculty of Medicine, Erciyes University, Kayseri, 38039, Turkey

^4^ GENKOK-Betül-Ziya Eren Genome and Stem Cell Center, Erciyes University, 38039, Kayseri, Turkey

^5^ Biomedical Engineering, Erciyes University, Kayseri, 38039, Turkey

^6^ Department of Nanomedicine, Houston Methodist Research Institute, Houston, TX 77030, USA

^7^ ERKAM-Clinical Engineering Research and Implementation Center, Erciyes University, 38030, Kayseri, Turkey

*** Corresponding Authors**

**Assoc. Prof. Omer Aydin**

Department of Biomedical Engineering,

Erciyes University, 38039, Kayseri, Turkey

**Phone:** +90-352-207-6666 / Ext: 32984

**e-mail:** [biomer@umich.edu](mailto:biomer@umich.edu); [omeraydin@erciyes.edu.tr](mailto:omeraydin@erciyes.edu.tr)

**S1. Calculation of Loading Efficiency of Quercetin and eEF2K-siRNA Molecules**

After the synthesis of the developed HNP particles was completed, standards of quercetin molecules were prepared at different concentrations (0.125, 0.25, 0.5, 1, and 2 mM) to determine the amount of quercetin bound to the AgNP core. By scanning the UV-Vis region (200-900 nm), the maximum absorbance wavelength of quercetin molecules was identified as 371 nm. The absorbance of each standard solution was then measured at 371 nm (Table 1). Based on the results, an absorbance-concentration calibration curve was plotted in GraphPad, and the linear regression equation and R² value were calculated (Supplementary Figure 1).

**Table 1:** Quercetin standards data obtained from UV-Vis spectrometer at 371 nm

| Quercetin Standard Concentration (mM) | Absorbance Value at 371 nm |
| --- | --- |
| 0.125 | 0.146167 |
| 0.250 | 0.280667 |
| 0.500 | 0.591521 |
| 1.000 | 1.206667 |
| 2.000 | 2.357167 |

**Supplementary Figure 1.** Calibration curve prepared with quercetin standard molecules

Following this step, the produced hybrid nanoparticles were centrifuged at 15,000 rpm for 30 minutes to precipitate the particles, and the supernatant containing unbound quercetin was diluted twofold and analyzed at 371 nm. The absorbance value obtained at this stage was recorded as “0.870176.” This value was then substituted into the calibration equation to calculate the concentration of free quercetin molecules, which was found to be 1.471566 mM. Given that the total volume of nanoparticles produced was 1.5 mL, the amount of unbound quercetin was calculated to be 2.207 µmol.

$$0.870176=1.184.5x-0.0016$$

$$x=0.735783$$

$$2x0.735783=1.471566 mM$$

$$(1.471566 mM)x(1.5 mL)=2.207 x{10}^{-6} mol=2.207 \mu mol$$

Initially, the concentration of the quercetin stock solution prepared for synthesis was 2.45 mM, and 2.04 mL of this stock solution was used, resulting in a total of 4.998 µmol of quercetin. Considering the total and unbound amounts, the loading efficiency of the quercetin molecules was calculated to be 55.84%.

$$(2.45 mM)x (2.04 mL)=4.998 x{10}^{-6} mol=4.998 \mu mol$$

$$Loading efficiency=\frac{Loadded amount}{Total amount} x100\%$$

$$\frac{\left( 4.998 \mu mol \right)-\left( 2.207 \mu mol \right)}{4.998 \mu mol} x100\%=\%55.84$$

**S2. Determination of the Optimal Ratio for Complex Formation Between eEF2K-siRNA Molecules and HNP Particles**

The optimal ratio for the complex formation between eEF2K-siRNA molecules and HNP particles was determined by keeping the concentration of eEF2K-siRNA constant while gradually increasing the concentration of HNPs. Each complex formed at different ratios was analyzed using agarose gel electrophoresis, following a protocol previously established in our laboratory. The ratio at which free eEF2K-siRNA molecules were no longer visible on the gel was considered to have formed the most efficient complexes this analysis, the 5:1 (NP:siRNA) ratio was identified as the most efficient, as all eEF2K-siRNA molecules in this ratio were bound to HNPs, preventing any visible fluorescence of free siRNA at the bottom of the gel (Supplementary figure 2-a).

Additionally, the zeta potential values of HNP and eEF2K-siRNA complexes formed at different ratios were measured to gain insights into the binding efficiency. Free siRNA molecules displayed a zeta potential of -27 mV, while the surface charge of the particles increased with the rising concentration of HNPs. The most efficient 5:1 (NP:siRNA) ratio was also the first to show a positive shift in surface charge, with an approximate zeta potential of +18 mV (Supplementary figure 2-b).


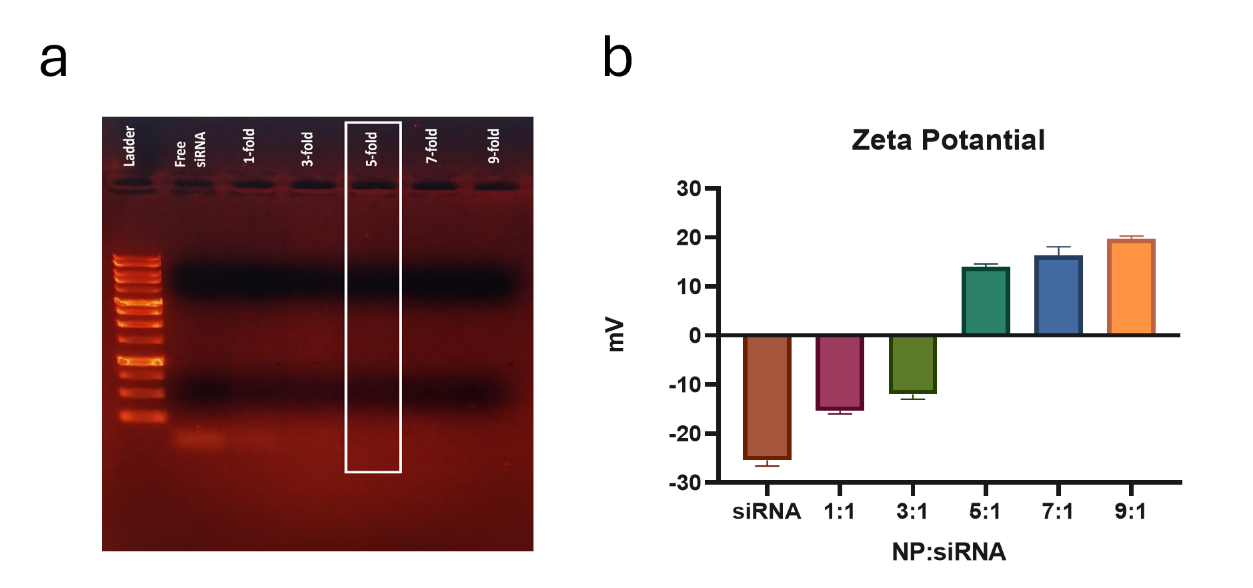


**Supplementary Figure 2.** (a) Agarose gel electrophoresis image obtained to determine the optimal ratio between HNP and eEF2K-siRNA molecules, and (b) zeta potential values measured for each complex formed.
